# Thirty years of geometric morphometrics: Achievements, challenges, and the ongoing quest for biological meaningfulness

**DOI:** 10.1002/ajpa.24531

**Published:** 2022-05-29

**Authors:** Philipp Mitteroecker, Katrin Schaefer

**Affiliations:** ^1^ Department of Evolutionary Biology, Unit for Theoretical Biology University of Vienna Vienna Austria; ^2^ Department of Evolutionary Anthropology University of Vienna Vienna Austria; ^3^ Human Evolution and Archaeological Sciences (HEAS) University of Vienna Vienna Austria

**Keywords:** ALSPAC, asymmetry, between‐group PCA, curse of dimensionality, human face shape, morphometrics, Procrustes

## Abstract

The foundations of geometric morphometrics were worked out about 30 years ago and have continually been refined and extended. What has remained as a central thrust and source of debate in the morphometrics community is the shared goal of meaningful biological inference through a tight connection between biological theory, measurement, multivariate biostatistics, and geometry. Here we review the building blocks of modern geometric morphometrics: the representation of organismal geometry by landmarks and semilandmarks, the computation of shape or form variables via superimposition, the visualization of statistical results as actual shapes or forms, the decomposition of shape variation into symmetric and asymmetric components and into different spatial scales, the interpretation of various geometries in shape or form space, and models of the association between shape or form and other variables, such as environmental, genetic, or behavioral data. We focus on recent developments and current methodological challenges, especially those arising from the increasing number of landmarks and semilandmarks, and emphasize the importance of thorough exploratory multivariate analyses rather than single scalar summary statistics. We outline promising directions for further research and for the evaluation of new developments, such as “landmark‐free” approaches. To illustrate these methods, we analyze three‐dimensional human face shape based on data from the Avon Longitudinal Study of Parents and Children (ALSPAC).

## 1 INTRODUCTION

About 30 years have passed since the foundations of geometric morphometrics were laid out. Thin‐plate spline deformation grids were published by Fred L. Bookstein in 1989, and the Procrustes method, earlier developed by Gower (1975) in a psychometric context, was extended to landmark data by F. James Rohlf and Dennis E. Slice in 1990 (but see also Boas, 1905 and Sneath, 1967 for earlier geometry‐based approaches). In his seminal 1991 book, Bookstein worked out a novel style of morphometric analysis by applying numerous multivariate statistical methods, including principal component analysis, multivariate regression, partial least squares analysis, and factor analysis to landmark data. This “orange book” also outlined methods to include curve information via semilandmarks and to disentangle symmetric and asymmetric shape variation. In 1993, Jim Rohlf and Les Marcus summarized these developments and coined them a “revolution in morphometrics.” The mathematical and statistical theory of shape analysis had been synthesized in the following years (Adams et al., 2004; Bookstein, 1996; Dryden & Mardia, 1998; Goodall, 1991; Goodall & Mardia, 1993; Rohlf, 1999; Small, 1996), based on the earlier work by David Kendall and others (Kendall, 1981, 1984). Since then, geometric morphometrics has been continually refined and has found countless applications in biological, anthropological, paleontological, medical, psychological, archeological, and industrial fields (for reviews see, e.g., Adams & Otárola‐Castillo, 2013; Bookstein, 2018; Cardini, 2020; Elewa, 2010; Halazonetis, 2004; Klingenberg, 2010; Lawing & Polly, 2010; MacLeod, 2018; Mitteroecker, 2020; Mitteroecker & Gunz, 2009; Schaefer et al., 2009; Slice, 2005; Wiley et al., 2005; Zelditch et al., 2012). The geometric morphometric toolkit has also been connected to other methodologies, including biomechanics (e.g., O'Higgins et al., 2019; Parr et al., 2012; Polly et al., 2016; Weber et al., 2011), systematics and phylogenetics (e.g., Adams, 2014; Klingenberg & Gidaszewski, 2010; Monteiro, 2013; Rohlf, 2002), image analysis (e.g., Mayer et al., 2014, 2017), quantitative genetics (e.g., Adams, 2011; Baab, 2018; Klingenberg & Leamy, 2001; Martínez‐Abadías et al., 2009; Pavličev et al., 2016; Schroeder & von Cramon‐Taubadel, 2017), genetic mapping (e.g., Klingenberg et al., 2001; Mitteroecker et al., 2016; Pallares et al., 2015; Varón‐González et al., 2019), evolutionary psychology and brain imaging (e.g., Walla et al., 2020; Windhager et al., 2012, 2018) as well as molecular and developmental biology (e.g., Arif et al., 2013; Buchberger et al., 2021; Hallgrimsson et al., 2015; Marchini et al., 2021; Martínez‐Abadías et al., 2018). Recent implementations of geometric morphometric methods into R and Mathematica facilitated analyses (Adams & Otárola‐Castillo, 2013; Dryden, 2021; Dryden & Mardia, 2016; Polly, 2017; Schlager, 2017).

**FIGURE 1:**
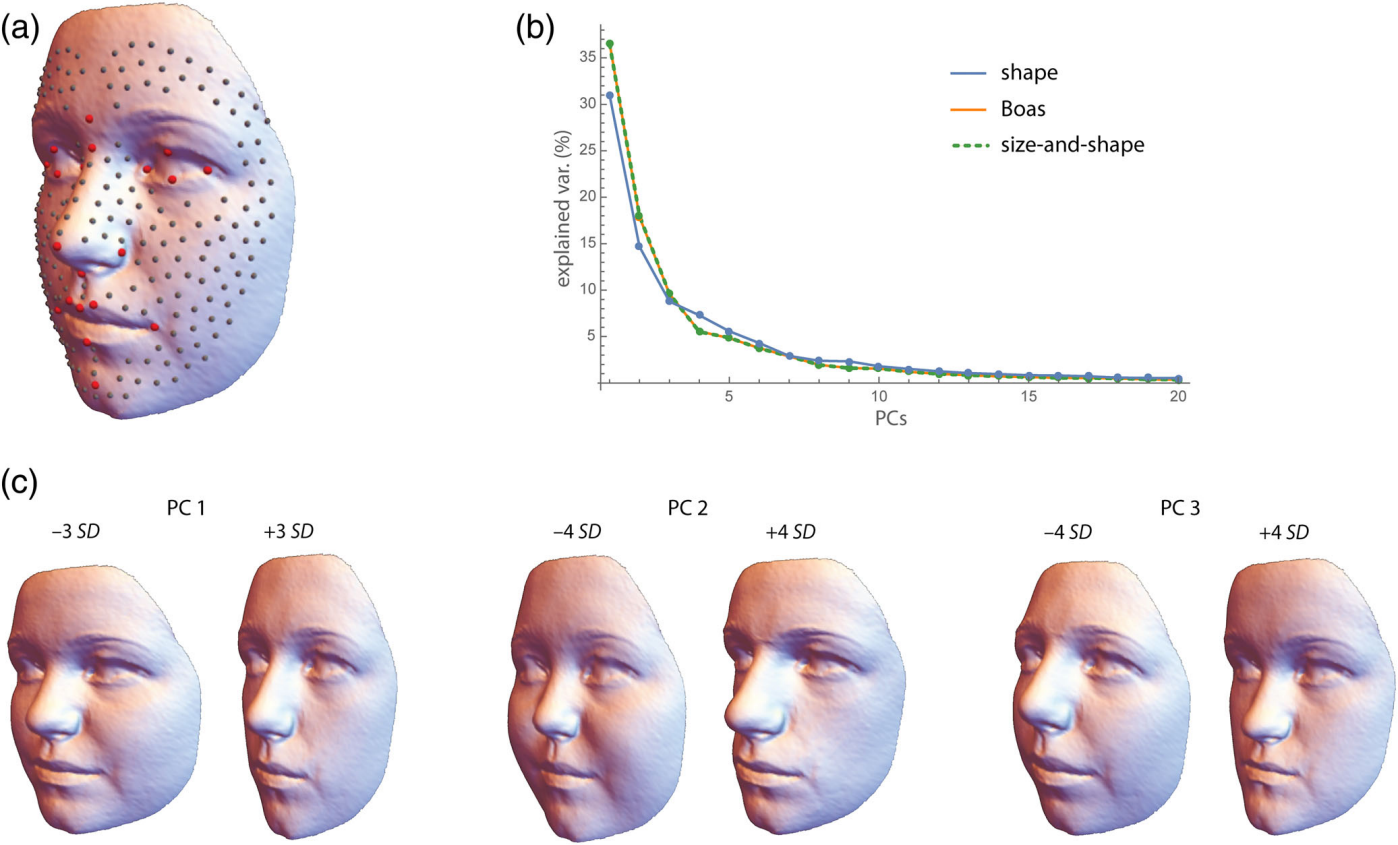
Our analyses of human face shape are based on a random sample of 100 female and 100 male white (based on parent's self‐reported ethnic background) adolescent individuals from the British cohort study ALSPAC (out of originally 15,454 pregnancies). Sex and date at birth had been obtained from the birth notifications. The average age in our sample was 15.5 years in both sexes (age range 14.7–16.8). Facial surface scans (Konica/Minolta laser scanners) along with the 3D coordinates of 21 anatomical facial landmarks were provided by ALSPAC (Toma et al., 2008). We created a template of 229 surface semilandmarks on one surface, which was warped onto all other surfaces based on the anatomical landmarks and then projected onto the surfaces. After sliding the semilandmarks by minimizing bending energy, the 200 configurations of 250 landmarks were superimposed by Procrustes analysis. (a) Sample mean shape with the 21 anatomical landmarks (red) and the 229 semilandmarks (gray). All facial depictions in this paper are the result of statistical analyses and do not reflect individual participants. (b) Scree plot for the shape coordinates (blue), the Boas coordinates (orange), and the size‐shape coordinates (green dashed). Only the first 20 dimensions are shown. (c) Visualizations of the first three principal components (PC) of face shape as warped surfaces corresponding to 3 or 4 standard deviations (SD) from the mean shape. PC 1 represents the overall width‐to‐height ratio of the face, PC 2 reflects the relative size of the nose and jaws, and PC 3 contrasts concave and convex facial profiles. Together, the first three PCs account for 54% of total shape variance

Here we review the “building blocks” of modern Procrustes‐based geometric morphometrics with an emphasis on recent methodological developments and current challenges, especially those resulting from the typically large number of morphometric variables. This paper is not meant as an introduction into geometric morphometrics; it addresses practitioners with some basic experience in morphometrics, but we avoid mathematical notation. It is also not a perfectly balanced representation of current morphometric practice as we emphasize the topics that we consider important, controversial, or promising. A main focus of this paper is the biological interpretability of morphometric and multivariate statistical analyses. We exemplify these methods and their interpretation by a series of analyses of three‐dimensional human face shape, using data from the ALSPAC study (Avon Longitudinal Study of Parents and Children; Boyd et al., 2013; Fraser et al., 2013); see Figure 1 and Acknowledgements for more details. We present these analyses and their results in the figures, basically as a picture story in parallel to the main text, which focuses on the methodological topics. Some specific details and technical comments are presented in endnotes.

We are fully aware of how morphometric analyses of human faces and bodies have been misused in the racist 20th‐century anthropology and of the problematic usages they can still offer today, such as the identification of ethnic minorities or the study of human remains (e.g., Hirst et al., 2018; Márques‐Grant & Errickson, 2017). In recent years, voices have become louder arguing that evolutionary and morphometric studies of human nature are altogether inappropriate. But studies of human morphological diversity are not only fundamental to the investigation of the human past, they are also indispensable for modern medical diagnostics and individualized treatment, forensics, textile design, and ergonomics (e.g., see Slice, 2005, and the references above). We believe that it is important to appreciate and study human diversity without intermingling biological differences with social or political narratives. Nonetheless, modern morphometric research *can* touch upon the boundaries set by our research policies and ethical guidelines. For instance, studies of human facial characteristics and personality, professional success, or sexual orientation are prone to be misused and must, if at all, be conducted with great care. Morphometric and statistical rigor can help to approach these challenges. For example, instead of superficial reports of statistical significance (which can always be achieved in sufficiently large samples), it is important to estimate and properly report effect sizes, such as average effects and explained variances, to show that even if such associations exist, *reliable prediction* of individual human behavior from face shape is not possible. In our opinion, the mere description and evolutionary interpretation of morphological differences between human groups is not problematic per se. For instance, Betti (2021) convincingly argued that understanding global variation in the form of the human pelvic canal can enhance and decolonialize obstetric care. In stark contrast, the application of morphometrics to identify politically persecuted groups is not compatible with scientific integrity. While several authors have addressed the history and political entanglement of early statistics and morphometrics (e.g., Bookstein, 1996; Cole, 1996; MacLeod, 2017; Stigler, 1999), a nuanced discussion of the role and potential misuse of modern morphometrics and statistical inference in anthropology is overdue but goes beyond the scope of this paper.

## 2 SIZE, SHAPE, AND FORM

All geometric morphometric methods are based on two‐dimensional or three‐dimensional landmark coordinates that represent biologically or geometrically corresponding point locations on the measured objects. Geometric morphometric methods differ in the way that shape (the geometric information independent of location, scale, and orientation) and form (geometric information independent of location and orientation, but not scale) of the landmark configurations are parameterized. The most common approach is based on a superimposition, or registration, of the configurations that standardizes for variation in position, orientation, and—if desirable—also scale. Other methods, such as Euclidean distance matrix analysis (EDMA; Lele & Richtsmeier, 1991), quantify form in a way that is invariant to changes in location and orientation in the first place; it does not require registration. This advantage, however, comes at the relatively high price of a complex geometry of shape or form space (Rohlf, 2000) and inefficient ways of visualization, both of which hamper the biological interpretation of results.

**FIGURE 2:**
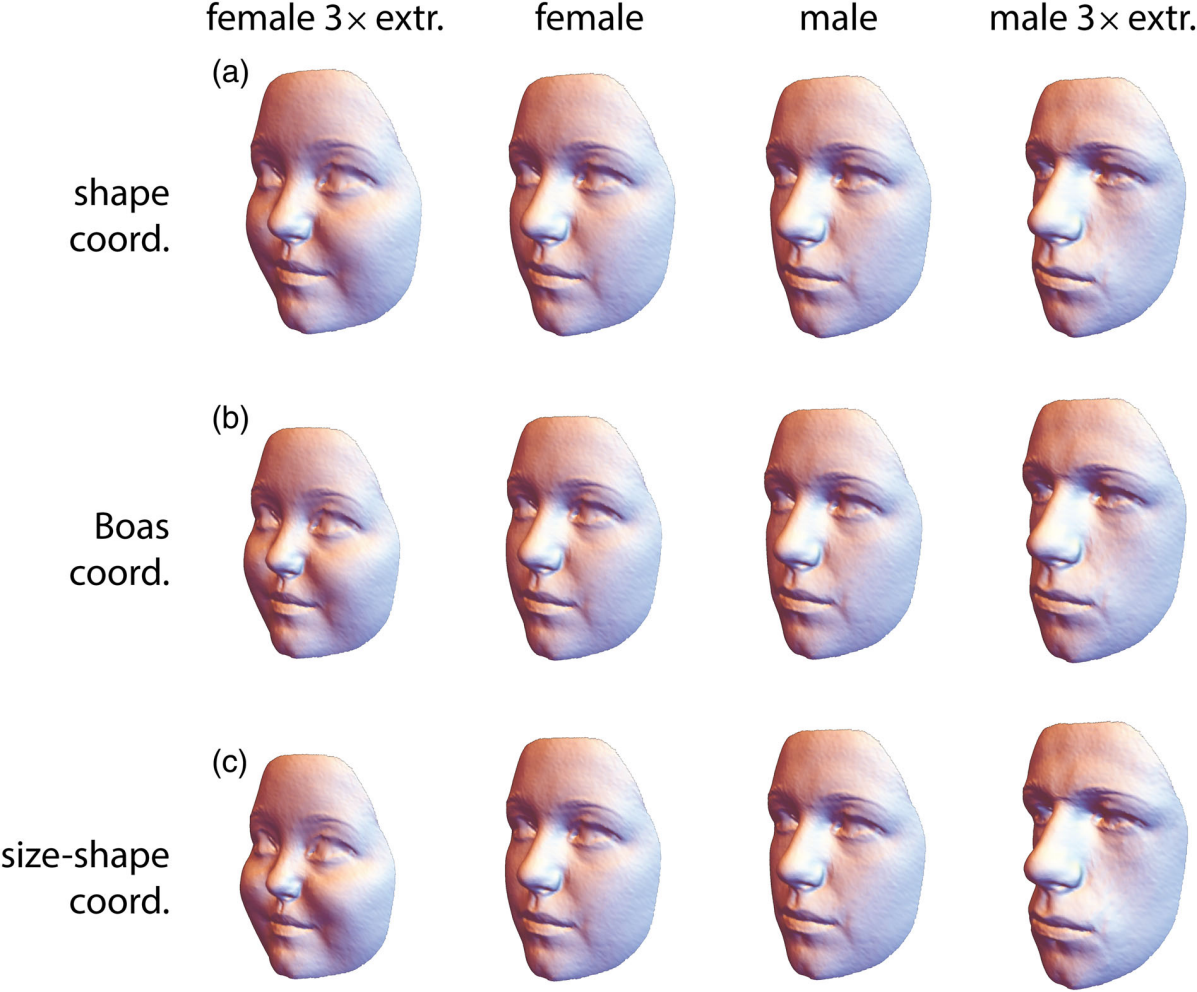
(a) Average shapes of the 100 female and 100 male face scans of the ALSPAC sample, along with three‐fold extrapolations of these differences (e.g., three times the mean difference between males and females was added to the female mean shape to yield the extrapolated male shape). (b, c) Average face forms as computed from the Boas coordinates and the size‐shape coordinates, respectively. Both methods yield very similar results and differ from panel a by depicting relative sizes also

The most common registration method in geometric morphometrics is Generalized Procrustes Analysis (GPA), which translates all configurations to the same centroid, scales them to the same centroid size (root summed squared distance of the landmarks from their centroid), and rotates them in order to minimize the summed squared differences between the configurations and their sample average (Rohlf & Slice, 1990). The translation and rotation steps in GPA are least squares approaches; the scaling to unit centroid size is geometrically convenient but does not minimize the squared differences between landmarks. For a discussion of different variants of GPA, including a full least squares approach, see Rohlf and Slice (1990) and Zelditch et al., (2012). A maximum likelihood version (Theobald & Wuttke, 2006) and a robust version (median‐based “resistant fit”; Slice, 1996) of Procrustes superimposition have been published, but they are not frequently used. After superimposition, the resulting shape coordinates can be statistically analyzed and the results can directly be visualized as shapes or shape deformations (Figure 1 & 2).

Standard GPA superimposes the configurations by reducing the differences between all measured landmarks, which is a clear improvement over earlier registrations that were often based on a more or less arbitrary choice of only two or three reference landmarks (e.g., the “Frankfurt horizontal” in cephalometrics). Nonetheless, some situations may warrant a registration on a subset of landmarks only, for instance, when some substructures of the studied anatomy are known to be more stable than others. Fornai et al. (2021) studied sacral vertebra shape in recent and extinct hominids and found a greater group separation when registering the configurations on the landmarks of the body of the first sacral vertebra only, as compared with a Procrustes registration based on all landmarks. It turned out that the relative size and shape of the sacral alae are more variable and also more species‐specific than those of the sacral body, but standard GPA intermingled these different signals.

Traditionally, most geometric morphometric analyses have been targeted at organismal *shape*, and the size of the studied structures is either ignored or analyzed separately. This makes sense if size is meaningless (e.g., because of unscaled images) or shows a different developmental or evolutionary behavior than shape. For instance, size is often much more variable than shape across species and more subject to phenotypic plasticity than shape within species. Furthermore, some functions may be primarily determined by the shape of an anatomical structure, not necessarily by its size. Nonetheless, other analyses may profit from a joint analysis of shape and size (i.e., form). When species differ both in size and shape, discrimination and classification studies are more successful when based on form rather than on shape. In our face sample, for instance, 87% of the individuals could be correctly classified as male or female based on form, but only 81% based on shape (leave‐one‐out cross‐validation using quadratic classification based on the first 10 principal components of the shape/form coordinates). Social perception of human faces, such as first impression formation and overgeneralizations, are strongly influenced by the shape of these faces, which is considerably correlated with facial size and stature (Butovskaya et al., 2022; Krenn, 2016; Schaefer et al., 2013; Windhager et al., 2011). Likewise, studies of growth, allometry, and heterochrony can be performed both in shape space and in form space (Cardini & Polly, 2013; Gerber et al., 2007; Klingenberg, 2016; Mitteroecker et al., 2004, 2005, 2013). In the absence of any prior expectations about size and shape variation, an initial exploratory analysis should also include the entire form information. Against the common tradition in morphometrics, we thus suggest starting with an analysis of organismal form, not only shape. Discarding size and focusing only on shape should be biologically justified (Bookstein, 2018; Klingenberg, 2016; Mitteroecker et al., 2013).

For a sample of *p* measured landmarks in *k* dimensions (2 or 3), GPA gives rise to *pk* shape coordinates, which are the landmark coordinates after standardizing location, scale, and orientation of the configurations. *Form coordinates* can be generated either by skipping the scaling step or by re‐multiplying the shape coordinates by centroid size. The ensuing superimposition standardizes location and orientation, but not scale. Bookstein (2018, 2021) referred to these coordinates as “Boas coordinates,” after Franz Boas who described them back in 1905. We will use this term here as well (see Klingenberg, 2016, for a review of different terminologies). A second, more common approach to derive form variables for landmark coordinates is to augment the *pk* shape coordinates by the natural logarithm of centroid size (log cs) as a separate variable, thus yielding *pk* + 1 form variables (Dryden & Mardia, 1998; Kendall, 1989; Mitteroecker et al., 2004). These variables have been termed size‐and‐shape coordinates, or simply size‐shape coordinates. For most analyses, the two approaches yield indistinguishable results, only for large size variation can the results deviate because size‐shape coordinates express size at a log scale whereas size is a linear factor in the Boas coordinates. In a multivariate analysis, the size of the configurations must be estimated indirectly from the Boas coordinates (e.g., as the first principal component of the data; see below), whereas it is an explicit variable in the size‐shape coordinates. Regression coefficients or principal component loadings for size can thus be directly inferred for size‐shape coordinates and represented via a biplot. But while the visualization of statistical results as forms is computationally straightforward for Boas coordinates, it requires a separate scaling step for size‐shape coordinates (Mitteroecker et al., 2013). The preferred kind of form variables thus depends on the focus of the analysis or the implementation in the software of choice, but the results will be similar for both approaches (see Figures 1b and 2b,c).

Centroid size is a convenient size measure in geometric morphometrics as it is based on all measured landmarks, and for small isotropic variation of the landmark coordinates around their sample mean (i.e., the same amount of uncorrelated variance in every direction, as a model of “pure noise”), centroid size is uncorrelated with shape. Under this so‐called Mardia‐Dryden distribution, the sample distribution is isotropic in shape space as well as in form space (both for Boas coordinates and size‐shape coordinates). This guarantees that pure noise in the landmark coordinates translates into pure noise in shape and form space.

Real data deviate from a Mardia‐Dryden distribution, and centroid size can be geometrically associated with shape features of interest. For example, because centroid size is computed from the *squared* distances between the landmarks and their centroid, landmarks along a circle have a smaller centroid size than corresponding landmarks along an ellipse of the same area (Bookstein, 2018). Hence, the centroid size of some neurocranial landmarks can vary and correlate with endocranial shape even if the endocranial volume was exactly the same for all specimens. Similarly, a wider gonial angle in a sample of mandibles might be associated with a larger centroid size because of the more elongated shape. Such correlations should not be misinterpreted as an allometric relationship; they are a geometric artifact. This does not preclude centroid size as a measure of scale in morphometric analyses (there is no “perfect” size measure that suits all purposes), but results should be interpreted carefully in this regard. Some analyses may also utilize other size measures that are more specifically targeted at the question or data at hand. For instance, endocranial volume and centroid size of endocranial landmarks strongly correlate and may often lead to similar results, but inferences about subtle differences in brain size may be more safely inferred from endocranial volume than from centroid size, especially in the presence of strong endocranial shape variation. Similarly, in a study of allometric shape variation in the human face, Mitteroecker et al. (2013) found that both facial centroid size and body height led to similar results for ontogenetic allometry as both measures are highly correlated throughout ontogeny. But for static allometry they yielded different results because adult facial size is influenced by factors unrelated to body height (e.g., body fat percentage).

In most morphometric samples, size varies more than shape. The first principal component (PC 1) of form variables thus is typically dominated by a combination of size variance and allometric shape variance, and the PC 1 scores may serve as a measure of “allometric size” (Bookstein, 1991, 2021). For form variables, PC 1 usually accounts for a larger fraction of total variance as compared with PC 1 of shape variables (Figure 1b). In a sample of multiple groups that differ both in size and shape, PC 1 can also be influenced by these group differences. In this case, size should better be estimated explicitly by centroid size or by the projections of the vectors of Boas coordinates on the mean vector, and allometry is best estimated by regressing the shape coordinates on a size measure, such as centroid size or body height. For multiple groups, different patterns of allometry can be compared as vectors in the first few principal components of shape or form space, but differences in length and orientation of these allometry vectors can also be assessed more directly (e.g., Schaefer et al., 2004; Simons et al., 2018). For more detailed reviews of allometry in geometric morphometrics see Mitteroecker et al. (2013), Klingenberg (2016), or Bookstein (2018).

## 3 VISUALIZATION AND STATISTICAL SIGNIFICANCE OF SHAPE AND FORM DIFFERENCES

For two‐dimensional landmarks, thin‐plate spline (TPS) deformation grids have proven very useful for visualizing shape differences, especially for identifying local shape features (Bookstein, 1991, 1997, 2000). Piras et al. (2020) reviewed several further methods for visualizing local shape deformations. Three‐dimensional shape or form differences often are best represented by a series of reconstructed shapes or forms, usually by deforming a mean landmark configuration along a given shape or form vector, such as a mean difference vector, principal component, or vector of regression coefficients. The biological interpretation is greatly enhanced by connecting landmarks into “wireframes” in an anatomically meaningful way, or by morphing (thin‐plate spline warping) a reference image or detailed surface representation (typically, the vertices of a triangulated reference surface) along with the actually measured landmarks, such as in Figure 2. This “morphing approach,” however, requires a sufficiently dense set or landmarks and often also semilandmarks (see below).

Linear extrapolation of differences allows one to display a given shape pattern at a greater magnitude, which can crucially facilitate the biological interpretation of subtle signals. For instance, the actual differences between female and male mean face shapes in Figure 2 are small and difficult to identify at the first glance, whereas the differences between the threefold extrapolations are obvious. Together, these four shapes effectively represent both the pattern and the magnitude of facial sex differences in this particular sample.

**FIGURE 3:**
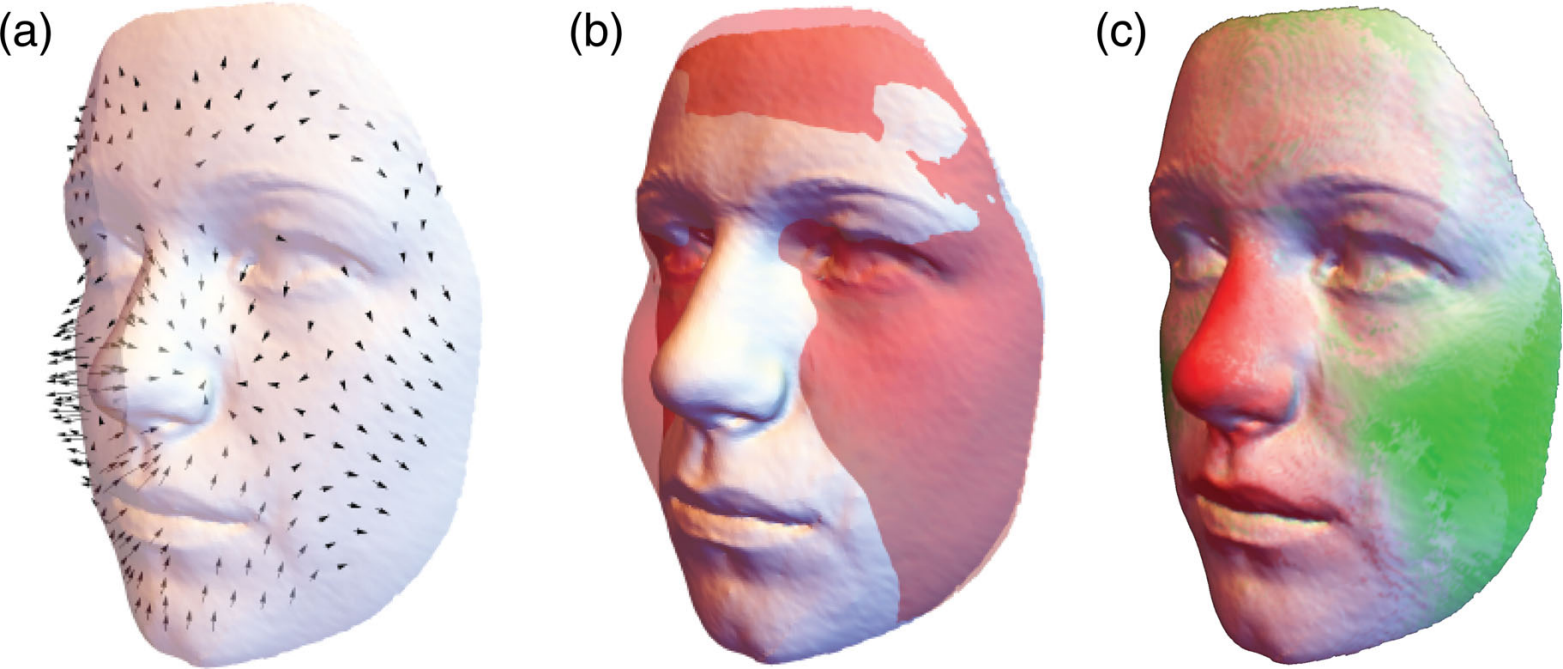
Visualization of average sex differences in face shape by (a) landmark displacement vectors (scaled by a factor of 4), (b) superimposed surfaces, and (c) a color map. The orthogonal differences between the two average facial surfaces were mapped onto a color gradient ranging from red (negative) to white (no difference) and green (positive). Compared to the surface morphs in Figure 2, these three figures are less effective in visualizing the shape differences. In particular, the color map is reducing three‐dimensional vectors to scalar quantities, thus omitting a considerable part of shape information. Whereas the males' larger nose and less defined cheeks can easily be recognized in panel c, the exact shape differences in these features as well as additional sex differences, such as comparably smaller eyes, a larger and more angular jaw and more prominent chin, cannot easily be inferred from these plots

Landmark displacement vectors and superimposed surfaces can be less effective, especially for visualizing complex 3D deformations, because not all parts of the visualized structures may be visible (Figure 3a,b). Unlike deformation grids and reconstructed shapes, landmark displacement vectors are subject to superimposition‐specific artifacts, such as the Pinocchio effect (Klingenberg, 2021; Richtsmeier et al., 2002). The reason is that shape is a relational property of *multiple* landmarks, and the coordinates of single landmarks should not be interpreted separately (see Section 9 and, e.g., Klingenberg, 2013).

Recently, it has become more common in geometric morphometrics to represent shape differences by color maps (Figure 3c). There are many ways of translating differences in landmark positions or surfaces into colors, such as distances orthogonal to the surface or between homologous locations, currents, differences in surface area, and magnitudes of local deformation or bending. The general weakness of color maps is that two‐ or three‐dimensional differences (2D or 3D vectors) are reduced to single quantities (scalars that are mapped onto a color gradient), which entails a considerable loss of information. Color maps are widely used in brain imaging, where the colors may really represent a scalar quantity, for example, cortical thickness or brain activity. But for visualizing shape or form differences, color maps often are insufficient. For instance, Figure 3c indicates an increase of nasal size and, to weaker magnitude, of jaw and brow ridge size, along with a narrowing of the cheeks. But none of the other details depicted by Figure 2a are visible here. We thus suggest using color maps only together with deformation grids or reconstructed shapes (e.g., Neubauer et al., 2020), if at all, when attempting to visualize shape or form differences.

Because of the intrinsically multivariate nature of geometric morphometric data and the difficulties of interpreting single landmark coordinates, statistical significance tests are generally multivariate and usually based on permutation tests (e.g., Adams & Collyer, 2015; Collyer & Adams, 2018). In most morphometric applications, however, multivariate null hypotheses of absolutely no effect do not align well with any biological hypothesis, and the alternative hypotheses (namely that at least one of the shape/form coordinates or linear combination of coordinates shows an effect) are not particularly informative. Most geometric morphometric studies thus heavily rely on multivariate exploratory methods and extensive visualization (see below for examples), thus requiring sufficiently large sample sizes (e.g., Cardini & Elton, 2007, and Section 5.2). Significance tests primarily serve to test against pure noise and to permit further exploratory analyses but rarely lead to relevant biological insights per se.

## 4 SLIDING LANDMARKS

Many modern morphometric studies have included semilandmarks to capture the geometry of curves or surfaces together with anatomical point locations. Typically, semilandmarks have only one coordinate that carries anatomical information, namely that orthogonal to the curve or surface. Their positions along the curve or surface are meaningless; they cannot be homologized across specimens based on anatomical criteria and—for the purpose of statistical analysis—must be estimated in a way that reduces artificial signals in the data resulting from arbitrary placement. Bookstein (1991, 1997) proposed the sliding landmark algorithm that “slides” the semilandmarks along tangents to the curve in order to minimize shape differences in the sample. Gunz et al. (2005) extended this approach to 3D surfaces. Reviews and comparisons of methods and software include Perez et al. (2006), Gunz and Mitteroecker (2013), Botton‐Divet et al. (2015), and Bardua et al. (2019). The initial placing of curve semilandmarks can often be performed manually, but the placing on surfaces usually requires some semi‐automated algorithm. For instance, semilandmarks can be placed manually or automatically on a reference specimen and are then warped to all other specimens based on the measured anatomical landmarks, which brings the semilandmarks close to each surface. Finally, they are projected onto the actual surfaces and subjected to the sliding landmark algorithm. We used this approach for the face data analyzed here. Alternative methods are discussed in Rolfe et al. (2021).

When sliding the landmarks, one can choose to minimize either the bending energy or the Procrustes distance between each configuration and the sample mean shape. Bending energy is a measure derived from the TPS algorithm and quantifies the magnitude of local shape deformation. Shape differences at small scales (i.e., of closely adjacent landmarks) have a higher bending energy than large‐scale differences. Procrustes distance is the square root of the summed squared distances between the corresponding landmarks in two superimposed configurations. Unlike for bending energy, the spatial configuration of landmarks does not affect the Procrustes distance because the squared differences are just summed up landmark by landmark. Bending energy measures only non‐affine (localized) shape differences; affine shape differences (linear scaling and shearing) are not reflected by bending energy.^1^ Nonetheless, when shape variation is small and the sliding of semilandmarks is sufficiently constrained by anatomical landmarks, both approaches typically lead to similar results. But in some situations, they can differ considerably. For instance, when affine shape variation is not sufficiently determined by anatomical landmarks, shape variance can even increase by reducing bending energy. In this case, Procrustes distance should be minimized (e.g., Bertl et al., 2016). In most situations, however, minimizing bending energy reduces—but not minimizes—total shape variance. Instead, it leads to the “smoothest” possible TPS deformation grids. Minimizing Procrustes distance does minimize total shape variance in the sample, but this may not necessarily imply a biologically plausible homology criterion (Gunz & Mitteroecker, 2013). By minimizing Procrustes distance the deviations are minimized for each landmark independently, therefore, a semilandmark can pass across an anatomical landmark, which may be at odds with biologically possible shape variation. A change in the sequence of landmarks is almost impossible to achieve by minimizing bending energy because such small‐scale shape changes are highly penalized. In some situations, however, this may be desirable. For example, in cephalograms (lateral cranial X‐rays) the anterior part of the mandibular ramus often projects above the posterior part of the palate, but we may not want that the position of the palate influences the sliding of semilandmarks on the mandible.

The sliding landmark algorithm involves multiple iterations, in each of which the tangent directions or tangent planes to the curve or surface are recomputed and the sample mean shape is updated. Whereas minimizing Procrustes distance leads to a convergence of the algorithm (i.e., at some point the mean shape stays unchanged and the semilandmarks do not slide any more), minimizing bending energy typically does not lead to convergence because the affine part of shape variation is not penalized. But in most situations, sliding reduces strongly after a few iterations and leads to a good correspondence of semilandmarks across configurations. Omitting the updating step of the mean shape can stabilize the algorithm.

If curves or surfaces are strongly bent, sliding along tangents can move the semilandmarks off the actual structure. This can be reduced by sliding only a given fraction of the computed distance along the tangents. As a result, the tangents are re‐estimated at smaller steps and trace the curvature more accurately. The increased number of iterations does not impose considerable computational costs. For all these reasons, it is advisable to carefully supervise the sliding process and to check if the slid semilandmarks stay close to the curve, cover the structures of interest, and represent biological or geometric correspondences.

Some authors have criticized the use of semilandmarks. For instance, Cardini (2020) wrote that “positions of the semilandmarks can be optimized, but they are fundamentally arbitrarily decided by an operator or an algorithm” (p. 514). This is incorrect: In contrast to entirely “homology‐free approaches” (see Section 9), semilandmarks must be placed on the *same* curve or surface (structures that are treated as biologically or geometrically homologous), for example, the neurocranium, and should be surrounded by homologous anatomical landmarks. Cardini criticized that, for instance, cranial semilandmarks close to the frontoparietal suture could slide on the frontal bone in some specimens and on the parietal bone in other specimens, and hence are not biologically homologous. But as explained above, the coordinates of semilandmarks along the surface are meaningless, and one cannot interpret the position of single semilandmarks, only the surface geometry that all semilandmarks describe *together*. If one cares about the frontoparietal suture, one must measure it by anatomical landmarks and/or curve semilandmarks (then, surface semilandmarks cannot arbitrarily slide across the suture, at least when minimizing bending energy). Clearly, based on surface semilandmarks only, no inference can be made about the bones constituting the measured surface; only the overall surface geometry can be interpreted. Whether or not this is sufficient depends on the research question. Cardini (2020) further argues that “none of the methods to slide the semilandmarks increases the accuracy of their mapping onto the underlying biological homology: […] none of them is based on a biological model, and the assumption of universal equivalence between geometric and biological correspondence is unverified, if at all verifiable.” (p. 513). Indeed, neither Procrustes distance nor bending energy are based on a biological model, as is the case for basically *all* other statistical metrics and methods. Moreover, many different and partly incongruent notions of anatomical, functional, geometric, developmental, and evolutionary homology have been employed in the biological literature. Therefore, all measurements, not only semilandmarks, need to be interpreted within a specific scientific context and with respect to its measurement system. In practice, sliding the semilandmarks often improves their correspondence and the interpretability of morphometric analyses, but a “universal equivalence between geometric and biological correspondence” is, of course, impossible.

## 5 THE GEOMETRY OF SHAPE AND FORM SPACE

The visualization and interpretation of statistical results as a two‐ or three‐dimensional geometry of *k* measured landmarks, i.e., as an actual shape or form, is a key strength of geometric morphometrics. But there is also another geometry that is often interpreted in morphometric analyses, namely the geometric relationships among the cases or groups in the 2*k*‐ or 3*k*‐dimensional shape/form space.^2^ Such interpretations comprise the clustering of shapes or forms into different groups (e.g., age groups, populations, species), the location of shapes or forms relative to such clusters (e.g., to infer taxonomic affiliation or evolutionary relatedness), and the geometry of developmental or evolutionary trajectories to infer processes such as allometric scaling, heterochrony, and developmental or evolutionary divergence.

The basic principle of interpreting such geometries appears to be obvious: Each shape or form is represented by a point in shape/form space; the distance between two points in shape/form space (Procrustes distance) is a measure of overall shape/form difference. Points along a linear trajectory in shape/form space represent a continual shape/form transformation; a bent in the trajectory indicates a changed transformation. The angle between two linear trajectories measures the deviation in the pattern of these two shape/form transformations. However, this classic rationale has been developed for small numbers of biologically meaningful and geometrically independent traits. In recent years, it became increasingly clear that such geometries are not always straightforward to estimate and to interpret for geometric morphometric data, especially when the number of landmarks is large and the distribution and density of landmarks across the organisms is arbitrary. Where is the problem?

### 5.1 Affine invariance

The standard interpretation outlined above treats all shape or form coordinates equally, regardless of whether they are x, y, or z coordinates, closely adjacent or distant landmarks, or whether they are located in a densely or loosely sampled area of the organism. Consider, for instance, a set of landmarks measured on primate crania: say 20 landmarks on the face and 20 on the neurocranium. Now consider four species, where species A and B have a very similar facial shape but differ in the neurocranium. Species C and D, by contrast, differ in the face but share a similar neurocranial shape. For the entire cranium, we will thus find similar Procrustes distances between A and B and between C and D. But what if we had measured 30 landmarks on the face and 10 landmarks on the neurocranium (which is actually more realistic)? This would weight the facial differences higher than the neurocranial differences, and based on overall Procrustes distance or its ordination via PCA we would conclude that A and B are more similar in cranial shape than C and D are. As the density of landmarks is often arbitrary (especially when using semilandmarks) or based on the available Type I and II landmarks, the “weighting” of the different anatomical regions by the number of landmarks may not be interpretable in a meaningful way. In other words, distances along different directions in shape or form space may not necessarily be comparable in total magnitude. These shape differences can of course be visualized and interpreted; only expressing their *magnitude* by a single quantity can be problematic if we are comparing “apples and oranges,” that is, qualitatively different shape deformations.

Similarly, in these standard interpretations all the shape or form variables are considered geometrically independent, that is, one assumes that one variable can, in principle, change without affecting other variables. This notion is reflected by orthogonal axes of data space.^3^ But shape or form variables typically are not geometrically independent. After Procrustes registration, the *pk* shape coordinates are geometrically linked (only the *pk*−4 or *pk*−7 dimensions of tangent space are geometrically independent; see Section 9). More importantly, the landmarks are biologically linked: spatially closely adjacent landmarks cannot vary independently. For instance, a covariance of, say, 0.001 between the two distant cranial landmarks nasion and lambda would be biologically more interesting than a covariance of 0.001 between nasion and glabella, which we expect to covary due to their adjacency anyway. But in standard analyses, they are all treated equally. Giving up the assumption of geometrical independence of variables implies that we give up the orthogonality of the axes in data space, which entails that angles of shape or form trajectories in different directions may not be comparable.

These arguments are uncommon in morphometrics and deeply unpleasant as they question the fundamental geometries on which many conclusions in the morphometric literature rest: distances and angles in shape or form space. If we take that seriously, at least as a worst case scenario, is there something left that we can safely infer from these geometries? Luckily, yes.

Increasing the density of landmarks in an anatomical region leads to a higher weighting of this region in multivariate distances and related statistics (e.g., Figure S1). Geometrically, this implies that the corresponding direction in an ordination (e.g., the first PCs) of shape space is “stretched” (i.e., approximately linearly scaled by the square root of the number of redundant variables; Bookstein et al., 2003; Huttegger & Mitteroecker, 2011). Similarly, changing the geometric dependence among variables is approximately equal to a change in the angle of the corresponding axes of data space. Therefore, at least in a first approximation, changes in the spatial density and subjective weighting of landmarks as well as changes in the geometric dependencies (e.g., spatial distance) among landmarks translate into linear scaling and shearing of a low‐dimensional ordination of shape/form space (Huttegger & Mitteroecker, 2011; Mitteroecker & Huttegger, 2009). As these weightings and dependencies often are either unknown or entirely arbitrary, we would consider only those conclusions meaningful that do not depend on any assumptions about these weightings and dependencies. In other words, only those findings are meaningful that are invariant to linear scaling and shearing of shape/form space, that is, invariant to any *affine* transformation of the space^4^ (Huttegger & Mitteroecker, 2011; Mitteroecker & Huttegger, 2009; Narens, 2002). Clearly, distances and angles are not invariant, but a number of other geometries are, such as incidence relationships. For example, points within a cluster remain in this cluster under all linear transformations, and two intersecting trajectories remain intersecting. Likewise, linear trajectories remain linear and parallel trajectories remain parallel under linear transformations. In addition, ratios of distances along the *same* direction are affine invariant. As a result, a point in between two other points remains in between after affine transformations. Finally, ratios of volumes in shape or form space are affine invariant, which implies that ratios of generalized variances are invariant (see Section 7).

In practice, this means that one cannot uniquely quantify the overall magnitude of the shape differences between species A,B and C,D in the above example because they deviate in different shape features or directions in shape space (but we can visualize and describe them). Any such quantification would be influenced by the more or less arbitrary decisions about the spacing and numerosity of landmarks. Only if two species E and F differed in the *same* cranial shape features as the species A and B (parallel directions in shape space) could we meaningfully compare their total magnitude. Likewise, findings that two shape trajectories are parallel or that one shape is in between two other shapes do not depend on any assumptions about number and spacing of landmarks. Within the first few PCs of shape space, also classification likelihoods and Mahalanobis distances are largely independent of the spacing of landmarks (Huttegger & Mitteroecker, 2011). This implies that statements about group overlap and separation, classification, and the proportionality of distributions (which underlies many null models in evolutionary quantitative genetics and multivariate significance testing) are meaningful. One cannot interpret absolute values and differences of multivariate or generalized variances, but *ratios* of generalized variances as well as relative eigenvalues based on the first PCs (Section 7) are largely independent of the spacing of landmarks (Bookstein & Mitteroecker, 2014; Huttegger & Mitteroecker, 2011). But all geometries based on distances and angles in different directions of shape or form space should be interpreted with great care; they implicitly assume that all landmark coordinates count equally and independently. If this assumption is biologically implausible, one should avoid interpreting these geometries. In our example, the landmarks are relatively evenly distributed across the face so that facial areas of the same size are equally weighted in the analyses, which is sensible but biologically also arbitrary (e.g., variation in the forehead is much higher weighted than variation in the lips).

A meaningful interpretation of multivariate distances along different directions usually requires a plausible biological or mechanical model. A simple classic example is Mahalanobis distance, which expresses the difference between two group means relative to the within‐group variance along this direction. For a polygenic quantitative trait, the expected amount of phenotypic change due to genetic drift is proportional to the genetic variance within the population. Consequently, for multiple, equally heritable traits, the between‐group covariance matrix is expected to be proportional to the within‐group covariance matrix (Lande, 1979). Distances between group means *relative* to the within‐group variance (i.e., Mahalanobis distances) thus relate to the probability that the population differences have evolved by drift. For multivariate normal distributions, Mahalanobis distance also relates to the likelihood of classification into this group. Mahalanobis distances and their ordination via canonical variate analysis (CVA) fell out of fashion in geometric morphometrics because they discard the Procrustes metric and require dimension reduction prior to computation. In specific evolutionary or classification contexts, however, it can be a useful affine‐invariant metric.

### 5.2 The curse of dimensionality

Another challenge arises from the sheer number of variables in geometric morphometrics. Consider a set of landmarks, measured on two specimens with a certain magnitude of independent measurement error for every coordinate. In addition to the actual anatomical form differences, these errors contribute to the Procrustes distance between the two configurations because it is highly unlikely that the *same* measurement error has occurred in both specimens. Measuring more landmarks on the same two specimens adds further measurement error and increases the Procrustes distance. Hence, the magnitude of shape or form differences in a sample is also a function of the number of measured variables, *even if no real anatomical differences exist*. This effect is often negligible for strong anatomical differences, but it can be relevant if the number of landmarks is large and the actual difference small. Similarly, consider a juvenile and an adult specimen of two species each, constituting two simple developmental trajectories. Even if both species show the same developmental shape transformation, the angle in shape space between the two trajectories increases with the number of measured variables because of increasing independent measurement error.

These are well‐known phenomena in multivariate statistics, often referred to as the “curse of dimensionality.” In high‐dimensional data spaces, all points are far apart and all angles are high.^5^ Clearly, this further challenges the interpretation of Procrustes distances and of angles between shape or form trajectories. It also complicates the estimation of multivariate distributions, classification likelihoods, cluster analysis, significance tests, and methods such as CVA, canonical correlation analysis, and relative eigenanalysis: The higher the dimension, *p*, of the data space (here the number of landmark coordinates) for a given sample size *n*, the more dimensions are “empty” or “almost empty” and the sample shows zero or very little variance in these dimensions. As a result, we cannot relate distances to the variance in these directions as this would entail a division through zero or a very small number (the inversion of a rank‐deficient or ill‐conditioned covariance matrix), and these statistics cannot be reliably computed.^6^

**FIGURE 4:**
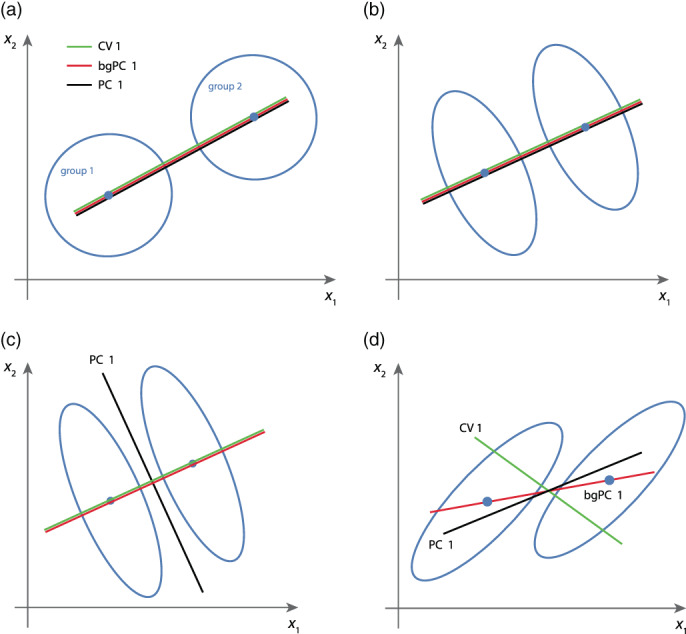
Schematization of principal component analysis (PCA), between‐group PCA (bgPCA), and canonical variate analysis (CVA). (a) When all groups have an isotropic distribution, the three methods yield the same results. (b) They also lead to the same result when the direction of the group mean differences is orthogonal or parallel to the major axes of within‐group variation, unless the within‐group PC 1 dominates over the group mean differences as in panel (c). (d) When the group mean differences are oblique to the major axes of within‐group variation, all three methods yield different results

**FIGURE 5:**
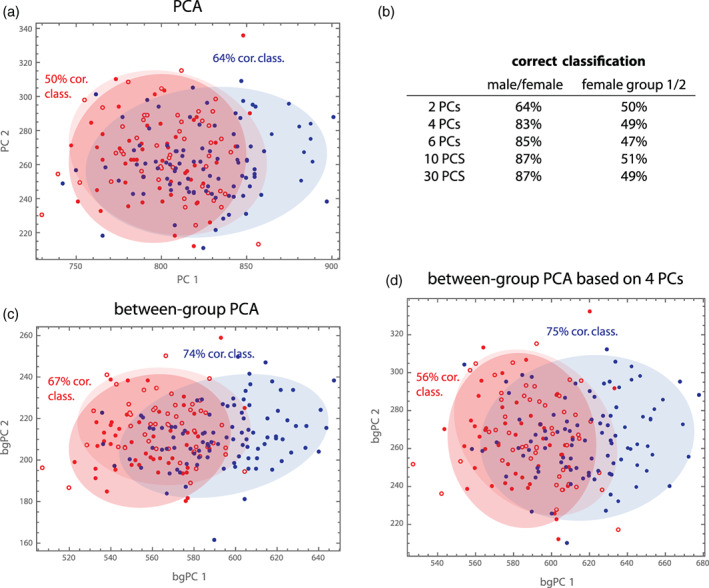
To illustrate properties of discrimination and classification, we consider three “groups” here: (1) the 100 males, (2) the first 50 females in the sample, and (3) the remaining 50 females. This random grouping of females shall represent two groups with the same statistical distribution. As variables we choose the Boas coordinates because males and females differ both in shape and size. (a) In a standard PCA, the two female groups completely overlap, whereas part of the male distribution deviates from the female distribution, as expected. Based on these two PCs, 64% of the cases are correctly classified as male or female (quadratic class., leave‐one‐out cross‐validation), and 50% of the females are classified as Group 1 or 2, as expected for completely overlapping distributions. (b) When using more PCs, over 80% of the cases can be correctly classified as male or female, suggesting that the first two PCs do not completely represent group differences. (c) Between‐group PCA better represents the separation between sexes (74% correct classification based on the two bgPCs), but also the two female groups slightly deviate in their distribution here, even though they should not. (d) When bgPCA is based on the first four PCs instead of all Boas coordinates, still 75% can be correctly classified as male or female, but the two female groups differ less

**FIGURE 6:**
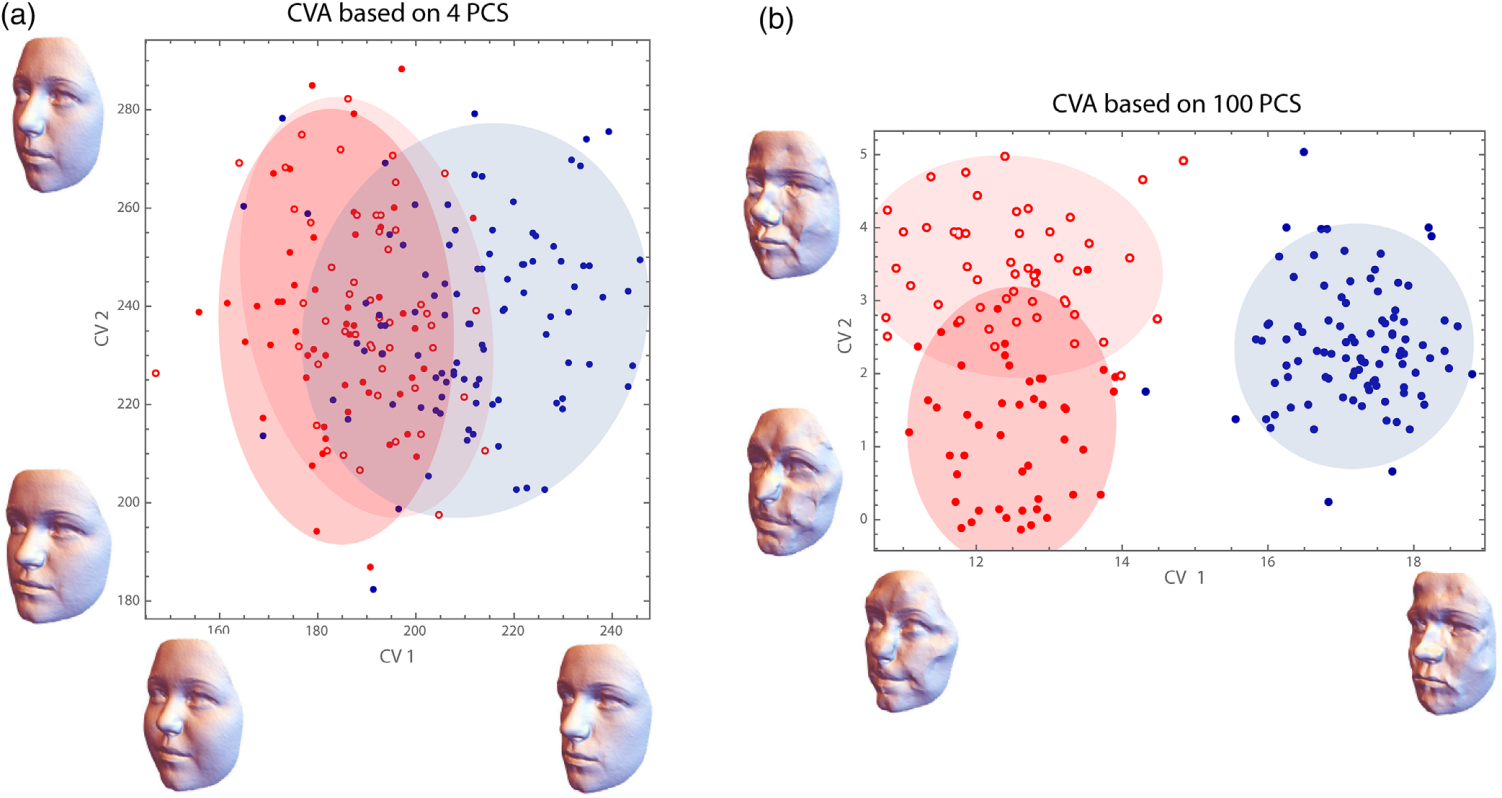
Canonical variate analysis (CVA) of the three “groups” from Figure 5. (a) When CVA is based on the first four PCs of the Boas coordinates, group overlap and classification rates are comparable to bgPCA. The CV axes can be directly visualized as form deformations (despite opposite claims in the literature; cf. Mitteroecker & Bookstein, 2011). CV 1 is similar, but not identical to the mean differences depicted in Figure 2 (e.g., the eye region does not strongly load on the CV). (b) When based on the first 100 PCs, which is still half the number of cases, CVA completely separates males and females, and also the two female groups differ strongly. This is an artifact resulting from the large number of variables. The form deformations corresponding to the CV axes are not interpretable; they represent just noise that happens to separate the groups. Clearly, new cases could not be successfully classified based on these features

In recent years, the geometric morphometrics community has intensely discussed these phenomena in the context of CVA and between‐group PCA (Bookstein, 2019; Cardini et al., 2019; Mitteroecker & Bookstein, 2011; Rohlf, 2021). In both methods, the separation of groups increases with the number of measured variables, even if the cases are sampled from the same distribution. Between‐group PCs are just the PCs of the group means, and the projections of the cases on these axes thus maximize the variance between the projected group means. For two groups, the single between‐group PC is just the multivariate vector through the two group means (Figure 4). As explained above, the distance between the group means tends to increase with the number of variables, even if the within‐group variance along this direction remains the same. As a result, the separation of the groups (i.e., the distance between the group means relative to the within‐group variance in this direction—the Mahalanobis distance) increases with the number of variables relative to the number of cases (Figure 5). This artificial group separation is even much more pronounced in CVA, where the variance between group means is maximized *relative* to the variance within groups. As increasingly many dimensions in data space are “almost empty” (i.e., with little variance) when *p* increases relative to *n*, CVA finds directions where the group means are far apart *relative* to the tiny within‐group variance (even if the group mean differences in this direction are also small and biologically irrelevant, e.g., just due to measurement error). As a result, CVA always separates groups, even along meaningless dimensions in data space, unless the sample size is much larger than the number of variables (Figure 6; Mitteroecker & Bookstein, 2011).

### 5.3 How shall we deal with this?

For two of the most common morphometric analyses, the computation of mean shapes and shape regressions, the number of landmarks does not impose a constraint because the averages or regression coefficients are computed for every shape coordinate separately and independently. PCA is based on all variables jointly but can also be computed if the number of variables exceeds the number of cases. In fact, PCA is the standard tool for dimension reduction in this situation. Furthermore, PCA is only based on the covariance matrix of the entire sample, not on any information about group affiliation, so that group separation in PCA does not generally increase with the number of variables, yet group separation is often underestimated in PCA (Figure 4).

Most other multivariate methods do depend to some degree on the number of variables relative the number of cases. Methods that maximize variances or covariances weakly depend on this ratio. For example, the artificial group separation in between‐group PCA is rather small for realistic geometric morphometric data that do show real group differences (Figure 6; see also Cardini et al., 2019). Similarly, the covariances that are maximized in partial least squares analysis (the singular values) depend on the number of variables (Mitteroecker & Bookstein, 2007; Bookstein, 2017), even though the shape patterns (singular warps) are relatively stable for realistic morphometric data. By contrast, methods that maximize a variance *relative* to another variance, such as CVA, canonical correlation analysis, relative eigenanalysis and related statistics, are more strongly affected by the number of variables. If *p* ≥ *n*, these methods cannot be computed from the original variables at all, but in our experience, reliable results require at least 5 to 10 times as many cases as variables. In practice, this always requires dimension reduction or regularization prior to these methods (also because shape coordinates are never of full rank^7^). For instance, CVA can be based on the first few principal components that capture the majority of variation (as inferred from a screen plot) instead of the shape coordinates.

**FIGURE 7:**
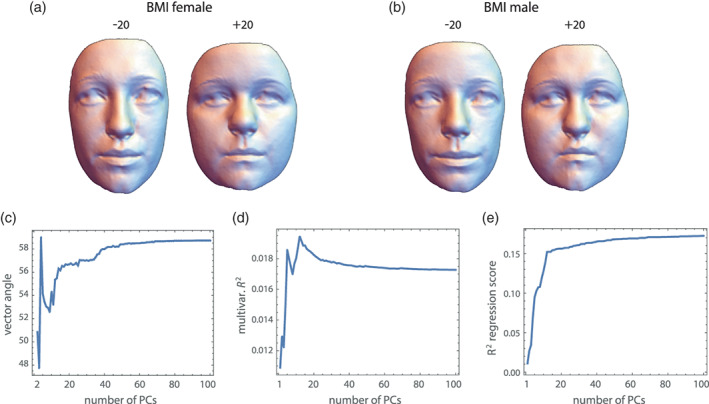
Effect of body mass index (BMI) on face shape, estimated by the regression of all 750 shape coordinates on BMI in females (a) and males (b). The reconstructed faces correspond to 20 units of BMI below and above the mean BMI value, which is a fourfold extrapolation of the actual BMI range in our sample. The BMI‐related shape pattern is very similar in both sexes, but the subcutaneous fat distribution in the cheek region differs slightly between the sexes. Despite these overall similarities, the angle between the vectors of regression coefficients in full shape space is 59°, which pools over all shape coordinates regardless of their spatial adjacency and may—incorrectly—be interpreted as a strongly divergent shape pattern. (c) Here this angle between male and female coefficient vectors is computed from an increasing number of PCs. The first 15 PCs (accounting for 86% of total variance) seem to contribute to the angle; adding more PCs slightly but continually increase the angle because they add further noise. (d) The multivariate *R* ^2^ (fraction of total shape variance explained by BMI) is as low as 1.7% in both sexes because it pools over all shape variables. Also here the first 10–15 PCs account for most of this explained variance. Adding further PCs decreases the multivariate *R* ^2^ because they are not really associated with BMI but inflate the total variance. (e) Another statistic of multivariate association is the *R* ^2^ between BMI and the regression scores (projections of the vectors of shape coordinates on the coefficient vector), which is 18% in females 16% in males. These statistics measure the association of BMI with the shape patterns depicted in panels a and b, not with *all* shape features. Again, the first 15 PCs constitute the signal, while all subsequent PCs inflate the *R* ^2^ because weak, random associations with BMI accumulate

The number of variables also affects the explained variance in shape or form regression. In Figure 7, for example, we studied the effect of body mass index (BMI) on face shape via shape regressions separately in both sexes. Clearly, BMI affects several aspects of face shape, especially the fat deposits in the cheeks, but most dimensions in shape space are unrelated to BMI. Averaging the explained variance over all shape coordinates thus resulted in a relatively small fraction of explained total shape variance (1.7% for our data). The larger the number of landmarks, the smaller would this fraction be because the number of dimensions in shape space without association increases. When computing this multivariate explained variance from an increasing number of PCs of face shape, we found that the first 10–15 PCs completely captured the signal (Figure 7d); adding more PCs weakly decreased the explained total variance because every further PC added a small amount of shape variance that is unrelated to BMI. An alternative summary statistic is the bivariate coefficient of determination, *R* ^2^, between the predictor variable and the regression score (the projections of the vectors of shape coordinates on the vector of regression coefficients, also referred to as “net partial predictor” by Bookstein, 1991), here the *R* ^2^ between BMI and the shape features depicted in Figure 7a,b. This yielded more realistic values of explained variance, 18% in females and 16% in males, because it quantifies the association only along the direction in shape space with maximal association, not along those many dimensions that are unrelated to the predictor variable. However, as in PLS, this correlation increases with *p*/*n*. For our data, after the signal was captured by the first 15 PCs, the *R* ^2^ increased slightly with every further PC (Figure 7e). For both statistics, therefore, absolute values of explained variance are difficult to interpret, but they can be compared across subsamples of similar size.

Especially when the number of landmarks is large, we suggest computing the abovementioned statistics (including bgPCA and PLS) as well as distances and angles in shape or form space also *after* dimension reduction, at least for comparison with those computed from all variables. Sample differences in explained variance can be clearer and more stable if computed from the first few PCs instead of the original shape/form coordinates. Some statistics, especially those related to prediction and classification, may be more effectively computed via regularization than dimension reduction. But dimension reduction by PCA usually is very effective in morphometrics because of the highly correlated variables, and it has the advantage that one can inspect which shape or form features contribute to subsequent computations.

If ordinary PCA does not suffice to represent group separation (Figure 4), we suggest using between‐group PCA or CVA on the first few ordinary PCs. In our experience, increasing the number of PCs for further computation usually increases group separation and classification success until all relevant factors of variation are captured (for the data sets that we analyzed, these were in the order of 5 to 15 PCs; see also Figure 5b). Thereafter, group separation does not increase considerably, until to the point when too much noise is added and the separation increases due to the sheer number of variables. Generally, statements about group overlap and separation should be supported by the cross‐validation of actual classification rates, for example, by leave‐one‐out or *k*‐fold cross‐validation of confusion tables, which can also guide the selection of PCs. Cardini and Polly (2020) and Thioulouse et al. (2021) also showed that the cross‐validation of bgPC scores largely alleviates the problem of spurious group separation.

More methodological research on dimension reduction in geometric morphometrics is needed, as this is such a crucial step for many analyses. PCA is a well‐understood and generally effective method, but it does not account for the specific nature and spatial structure of shape and form coordinates; it is not specifically designed for geometric morphometric data. When the signals of interest are known to be of a particular spatial scale, partial warps or principal components weighted by bending energy (Bookstein, 1991) may be possible. Bookstein (2015) proposed the use of “relative intrinsic warps,” which are the relative eigenvectors of the non‐affine part of shape with respect to bending energy. Instead of detecting the shape features that vary most (as in ordinary PCA), they detect the shape features with the maximal variance relative to the variance expected at this spatial scale under a self‐similar shape distribution^8^ (see Section 9). These directions of research seem promising, and there is ample space for new innovations, too.

## 6 HOW MANY LANDMARKS DO WE NEED?

After having finished and interpreted a geometric morphometric analysis, one may be tempted to think that it would have been possible to arrive at the same conclusions with fewer landmarks. The problem is that before knowing the results, it can be difficult to say *which* landmarks are the important ones. Questions, disagreements, and studies about the necessary number of landmarks, and especially of semilandmarks, have fueled numerous discussions in the geometric morphometrics community (e.g., Cardini, 2020; Evin et al., 2020; Goswami et al., 2019; Oxnard & O'Higgins, 2009; Rolfe et al., 2021; Watanabe, 2018).

Designing a landmark scheme is a crucial step in any morphometric study that requires time, biological knowledge, and a careful inspection of multiple specimens to gauge the range of variation to be represented. The biological or geometrical homology criteria underlying the landmark definitions are key to the interpretation of results (Bookstein, 1991; Oxnard & O'Higgins, 2009). Often, the major patterns of variation and group separation can be inferred from relatively small sets of anatomical landmarks, particularly if the relevant shape features are known a priori and the landmarks can be chosen accordingly. But if the features are unknown or of small spatial scale, a denser set of landmarks is necessary.

In general, the number and spacing of landmarks should be determined by the variation and spatial scale of interest, but also by the aim of the study. For a classification study, anatomical details are irrelevant as long as classification is successful. A functional or comparative study, by contrast, may depend on a more detailed representation of anatomical structures. Fossil reconstruction by geometric morphometric methods typically requires a very dense set of landmarks and semilandmarks (Benazzi et al., 2011; Freidline et al., 2012; Gunz et al., 2009). Biological background knowledge, visual inspection of specimens or preliminary studies inform about locations that vary strongly or at small scales, which should be captured by more landmarks.

An important strength of geometric morphometrics is the effective visualization of results, which allows for powerful exploratory studies. Based on such visualizations, one can identify features that one did not expect and would not have explicitly measured. Detailed shape or form visualizations typically require a sufficiently dense set of landmarks and often also semilandmarks. As a rule of thumb, one can try to imagine if connecting the landmarks by lines or polygons would sufficiently represent the structure at the intended level of detail.

Increasing the number of landmarks typically adds shape features of small scale, that is, partial warps with high bending energy. In most morphometric analyses, the first few principal components are dominated by large scale shape variation (this is why increasing the spatial density of landmarks may have little effect on the first few PCs), but one can focus on particular levels of spatial scale by a PCA weighted by bending energy (“relative warps,” Bookstein, 1991), or one can perform a PCA of shape variation relative to the variation expected for the spatial scale under a self‐similar shape distribution (“relative intrinsic warps,” Bookstein, 2015, 2018).

In our opinion, it is often advisable to start out with a large landmark set. It is easy to skip irrelevant or unreliable landmarks throughout the analysis, but it is time‐consuming or impossible to add further landmarks after the main data collection. It has been suggested that the statistical challenges arising from a large number of variables outweigh the advantage of spatial resolution in geometric morphometrics. However, the “curse of dimensionality” is gradual and already arises from a relatively small set of landmarks. In particular, methods that involve the inversion of a covariance matrix always require prior dimension reduction or matrix regularization, regardless of the number of landmarks, because shape coordinates are never of full rank and most sample sizes do not sufficiently exceed the number of shape or form coordinates. It has also been argued that a dense landmark set increases spatial autocorrelations and complicates studies of morphological integration and modularity (e.g., Cardini, 2019; Goswami et al., 2019). But again, spatial autocorrelations are always present in morphometric data and should be modeled appropriately if covariances among shape or form coordinates are to be interpreted (see Section 9).

## 7 QUANTIFYING AND COMPARING FORM VARIATION

Measures of phenotypic variation among individuals within species or populations have been important in diverse scientific contexts as they can reflect genetic and environmental heterogeneity, developmental instabilities as well as past regimes of stabilizing selection. Contrasting phenotypic variation *among* population mean forms with individual variation *within* these populations can reveal traces of divergent or stabilizing selection among populations (e.g., Bookstein & Mitteroecker, 2014; Grabowski & Roseman, 2015; Marroig & Cheverud, 2004). In medical contexts, understanding “normal” variation of traits is often pivotal to identify pathological forms. However, for highly multivariate data, such as in geometric morphometrics, quantifying and comparing variances can be challenging. Pooling variance over many variables, regardless of their spatial and anatomical relationships, can hinder biological interpretation for the reasons explained in Section 5. It is also problematic because different shape or form features often have very different variational properties, which are concealed by simple summary statistics.

The most common statistics to quantify the total magnitude of variation in multivariate data are the total variance (sum of all variances, or equivalently, the sum of the variances of the PCs) and the generalized variance (determinant of the covariance matrix, or equivalently, product of the variances of the PCs). The generalized variance can only be computed based on the first few PCs (otherwise one would multiply by zeros), but ratios of generalized variances (e.g., the ratio of the generalized variances of two populations) are affine invariant and thus largely independent of landmark spacing (Huttegger & Mitteroecker, 2011). Both statistics, however, pool over all variables and do not allow for any exploration of different variational dynamics. By contrast, PCA decomposes the data into different linear combinations (shape/form features) with successively lower variances, but these variances are not necessarily interpretable because they crucially depend on the spacing of landmarks. Consider, for instance, a sample of crania with comparable magnitudes of variation in the face and the neurocranium. If the face had more landmarks than the neurocranium, facial variation would more strongly impact measures of total variation and it would also dominate PC 1. More landmarks on the neurocranium than on the face would lead to the opposite result. For the same reason, large‐scale patterns of shape variation (e.g., the overall width‐to‐height ratio of the face) typically constitute PC 1 just because many correlated variables load on this component (compare Figure 1c).

**FIGURE 8:**
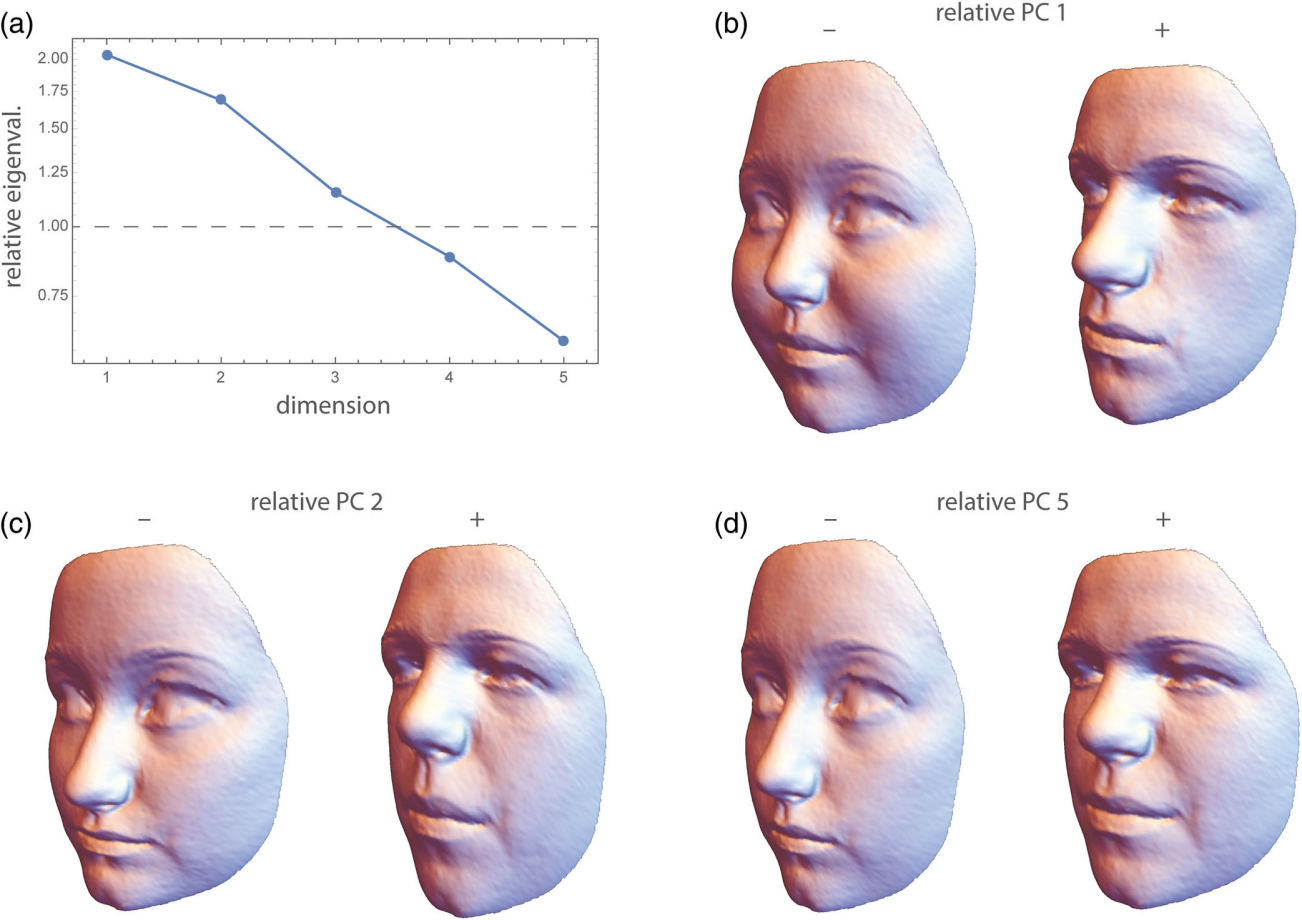
Total variance in face shape is about the same in both sexes (0.0014), but the generalized variance in males is 2.2 times higher than that in females. Both statistics, however, conceal the fact that different facial features differ in their variational properties. (a) Eigenvalues of males *relative* to females, that is, the maximal variance ratios in face shape, computed from the first five PCs of the data (plotted on a log scale). The first relative PC is about twice as variable in males as in females. The associated shape pattern (the first relative eigenvector shown in panel b) mirrors the sex differences depicted in Figure 2. Due to the earlier completion of puberty in females, this may reflect a higher variance in sex‐hormone related face development in males of this age group. (c) The second relative PC is still 1.7 times more variable in males and mainly reflects the height of the lower face relative to that of the upper face—a pattern generally attributable to allometric growth. (d) Relative PC 3 (chin protrusion) and PC 4 (forehead protrusion) have similar variances in both sexes (not shown), whereas the last relative PC (relative PC 5; a combination of overall width‐to‐height ratio and relative midface size) is 1.6 times as variable in females compared with males, maybe due to a slightly higher variance in BMI. Note that these variance ratios are affine invariant and thus largely independent of landmark spacing; their product equals the ratio of generalized variances

Unlike variances, variance *ratios* are invariant to linear scaling. For instance, a statement such as: “Population A has a 30% higher variance in body mass than population B” does not depend on whether body mass is measured in grams or kilograms, even though each of the two variances do depend on the measurement scale. Instead of maximizing variance as in PCA, we may thus want to find linear combinations that maximize the variance ratio between two groups. This approach is called “relative eigenanalysis” or “relative PCA” (Flury, 1985; Bookstein & Mitteroecker, 2014). As relative PCA involves the inversion of a covariance matrix, it requires dimension reduction or regularization prior to computation. Bookstein and Mitteroecker (2014) and Le Maître and Mitteroecker (2019) presented morphometric applications of relative PCA to investigate the generation and canalization of variance during development, population differences in variance patterns, and also an example of medical classification. Figure 8 shows an application to our face data.

Another field of application is in evolutionary biology. As a standard null‐model of neutral evolution, we expect the variance between population means to be proportional to the genetic variance within the ancestral population (often approximated by the pooled phenotypic within‐population variance of the descendant populations). For multivariate data, this implies that deviations from proportionality of the between‐group covariance matrix and the within‐group covariance matrix can be indicative of divergent or stabilizing selection. Several authors (e.g., Ackermann & Cheverud, 2004; Marroig & Cheverud, 2004; Martin et al., 2008) published significance tests of the proportionality of these two matrices but could not disentangle the features that drive the deviation from proportionality. When applied to the between‐population and within‐population covariance matrices, relative PCA provides a tool for exploring these features. For instance, using classic morphometric data, Bookstein and Mitteroecker (2014) found that facial height relative to neurocranial breadth has likely been subject to divergent selection in human populations because it was the feature that varied most between populations *relative* to the variance within populations (relative PC 1). By contrast, the relative size of the nasal cavity was the feature with minimal between‐population variance relative to the within‐population variance (last relative PC) and is thus likeliest to have been under stabilizing selection, if any trait was.

The sum of squares of the log relative eigenvalues serves as an affine‐invariant metric for covariance matrices, which has been used to study developmental and evolutionary changes of variance–covariance patterns (Gonzalez et al., 2011; Mitteroecker & Bookstein, 2009), but also other metrics have been proposed (Aguirre et al., 2014; Dryden et al., 2009).

Apart from univariate and multivariate variances, further metrics of “disparity” have been used in ecology and evolutionary biology to assess the morphological diversity (sometimes termed “morphospace occupation”) of different species or higher taxa (e.g., Guillerme et al., 2020; Hopkins & Gerber, 2021; Zelditch et al., 2012). These metrics include means and (trimmed) ranges of pairwise Procrustes distances. While such statistics can be useful in certain contexts and tend to be more robust against outliers than variances, their statistical properties cannot easily be linked to quantitative genetic theory and other multivariate methods.

## 8 DISENTANGLING SYMMETRIC AND ASYMMETRIC FORM VARIATION

For bilaterally symmetrical shapes, geometric morphometrics allows for a disentangling of symmetric and asymmetric shape features and a corresponding decomposition of total shape variation (Benítez et al., 2020; Bookstein, 1991; Klingenberg & McIntyre, 1998; Mardia et al., 2000; Schaefer et al., 2006). This applies both to object symmetry (e.g., the human face) and to matching symmetry (e.g., left and right hands). These methods have also been extended to more complex patterns of symmetry, such as rotational and nested symmetries (e.g., Klingenberg, 2015; Savriama & Gerber, 2018; Savriama & Klingenberg, 2011). For bilateral symmetry, the basic principle is to contrast a shape with its relabeled reflection. A perfectly symmetric shape is identical to its reflection. The difference vector between a shape and its reflection describes the object's asymmetry. The corresponding Procrustes distance (the length of this vector) can be interpreted as the total magnitude of shape asymmetry, and the angle in shape space between two asymmetry vectors as the deviation in asymmetry pattern, subject to the caveats mentioned in Section 5. The classic asymmetry literature further distinguishes between directional asymmetry (the average pattern of asymmetry in a sample) and fluctuating asymmetry (the individual deviations of asymmetry from the average pattern). Fluctuating asymmetry has been linked to both environmental (diet, climate, toxins) and genetic (aneuploidy, heterozygosity, inbreeding) stressors and thus is widely used as a measure of developmental instability (e.g., Graham & Özener, 2016; Klingenberg, 2015; Schaefer et al., 2006). Directional asymmetry, by contrast, often reflects genetically determined developmental differences between left and right body sides (Klingenberg, 2015).

The average of a shape and its relabeled reflection is a perfectly symmetrical shape. By exploiting this property, one can “symmetrize” the landmark configurations and thus remove asymmetric variation, including asymmetric noise, from the data. For small samples, this can lead to more regular visualizations and lower *p*‐values (because “unexplained” variance is reduced). If asymmetry studies are based on semilandmarks, as in our face example, the mean shape in the sliding landmark algorithm must be symmetrized to remove asymmetry resulting from an asymmetric initial placement of semilandmarks. As symmetric shape components usually vary much more across individuals than asymmetric shape, the first principal components of shape tend to capture symmetric variation only. For our face data, asymmetric variation accounted for only 7.8% of total shape variance, and the first PCs as well as the mean differences and shape regressions were all approximately symmetric (Figures 1, 2, 3).

**FIGURE 9:**
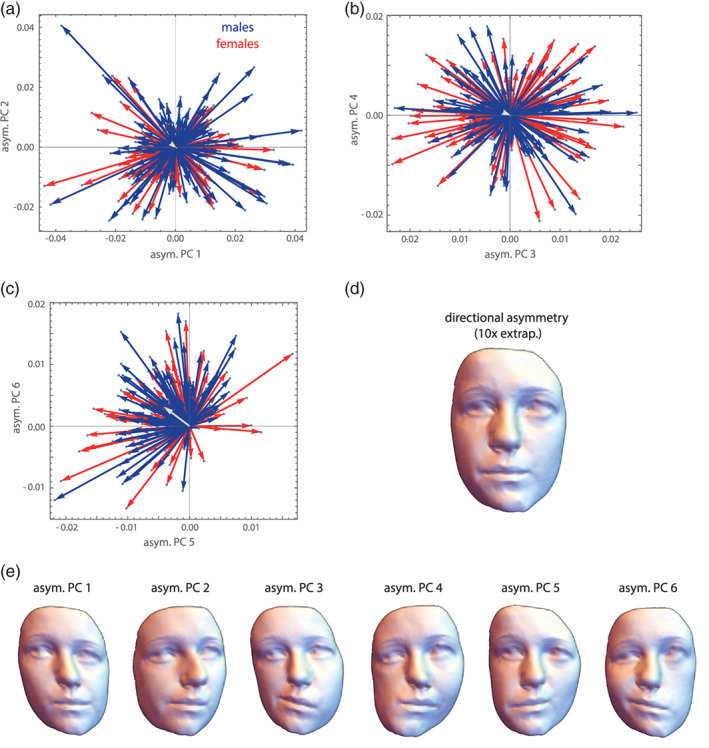
Asymmetry analysis of face shape. (a–c) The first six principal components of the asymmetry vectors maximize the variance around zero (not the mean as in ordinary PCA), which corresponds to perfect symmetry. Asymmetry vectors in shape space can differ in orientation (spatial asymmetry pattern) and length (total magnitude of asymmetry), but males and females do not seem to differ for any of these components here. The light gray vector represents directional asymmetry, that is, the sample average of all these asymmetry vectors. In contrast to the first four components, PCs 5 and 6 show a directional trend. (d) The visualization of directional asymmetry by a tenfold extrapolation of the difference between the average face shape and its relabeled reflection reveals a tendency for a larger left facial side compared to the right side, which was also reported by Dane et al. (2002, 2004). (e) Visualizations of the first six PCs of asymmetry as deviations from perfect symmetry (shown only in one direction)

In contrast to symmetric shape features, the asymmetry vectors have a natural origin: a vector of all zeros indicates perfect symmetry. In an ordination analysis of shape asymmetry, it is thus useful to represent the individual asymmetries as vectors from the origin (not just as points as in standard PCA), so that both pattern and magnitude of asymmetry are comparable (Figure 9). Standard principal components maximize the variance, that is, the average squared deviation from the mean. But for asymmetry vectors it is more effective to maximize the average squared deviations from zero (perfect symmetry), which can be achieved by a singular value decomposition of the uncentered data matrix (Neubauer et al., 2020). This way, the directional asymmetry patterns of face shape were captured by the fifth and sixth dimension of the modified PCA (Figure 9c). In an ordinary PCA of the asymmetry vectors, which maximizes only the fluctuating component of asymmetric shape variation, no directional pattern was visible among the first 15 dimensions of our data.

Both the symmetric and asymmetric shape features can be regressed on other variables. Associations *between* symmetric and asymmetric shape features can be studied via PLS or by regressing symmetric shape features on the magnitude of asymmetry, for example, to assess if certain shapes are particularly prone to some directional asymmetry patterns or higher magnitudes of asymmetry, respectively. For our face data, for instance, we found that more projecting faces with a longer nose tend to show a higher magnitude of total asymmetry as compared to flatter faces. But as symmetric and asymmetric components of shape constitute orthogonal subspaces of tangent shape space, they cannot be compared by methods such as relative eigenanalysis, which apply only to the comparison of two groups for the *same* variables. Similarly, angles between shape vectors (e.g., PCs or vectors of regressions coefficients) of symmetric and asymmetric shape components cannot directly be compared because they are in orthogonal subspaces.

In the classic as well as in the geometric morphometric literature it is common to contrast the magnitudes of fluctuating and directional asymmetry via a sum‐of‐squares decomposition (Procrustes ANOVA) and to statistically test a null hypothesis of zero directional asymmetry (Klingenberg, 2015; Klingenberg & McIntyre, 1998; Mardia et al., 2000). For our face data, directional asymmetry accounted for only 4% of the total sum of squares in both sexes, but a H_0_ of no directional asymmetry was clearly rejected. As usual, however, such a scalar summary of complex multivariate data can conceal a more nuanced picture. For example, the asymmetric shape feature depicted in Figure 9d showed a very strong directionality (compare Figure 9c), whereas all other shape features showed virtually no directional asymmetry (the mean vectors in Figure 9a,b have a length of almost zero).

Because fluctuating asymmetry is often subtle, it can be difficult to distinguish it from measurement error, which also tends to be asymmetric. Many studies of asymmetry thus involve repeated measures of at least some of the measured specimens. Procrustes ANOVA can then be used to decompose symmetric variation, asymmetric variation, and measurement error (Klingenberg, 2015).

## 9 MORPHOLOGICAL INTEGRATION, MODULARITY, AND SPATIAL SCALE

Different anatomical structures of organisms develop, vary, and evolve jointly. Biologically, this is almost a truism: Because of developmental interactions among adjacent tissues, growth factors with widespread effects, and the expression of genes in multiple tissues and time periods, different parts of an organism do not develop in isolation. Statistically, we observe that the dimensions of such parts covary across individuals, which in turn affects how a population responds to natural selection of one or more of these parts. Studies of these relationships are usually performed under the heading “morphological integration,” after the eponymous book by Olson and Miller (1958). The more modern literature has also focused on “modularity,” that is, the fact that these associations are not uniform across traits: some structures are less integrated (more modular) than others. It turned out, however, that a biologically meaningful geometric morphometric analysis of these intuitively appealing concepts is quite challenging. For instance, Olson and Miller interpreted the raw correlation coefficient between two measured traits as the strength of their integration, but this interpretation does not hold for larger sets of interlandmark distances or shape coordinates. In particular, a correlation of zero does not serve as a meaningful null model of no integration.

The first reason of this—perhaps unintuitive—fact is of a geometrical nature. Consider, as a simple example, four 2D landmarks that vary isotropically around a mean shape. In other words, the eight raw landmark coordinates are all uncorrelated and have the same variance, which is the typical null‐model of pure noise in geometric morphometrics (the Mardia‐Dryden distribution). The form of these configurations has only five degrees of freedom because we ignore variation in the overall location (2 df) and orientation (1 df), whereas shape has four degrees of freedom as we additionally ignore variation in scale (1 df). If we consider the six interlandmark distances for each configuration, we find all the distances that share a landmark to be correlated even though the landmark coordinates are all uncorrelated (these correlations reach a magnitude of about *r* = 0.35, regardless of the amount of isotropic noise). Similarly, after Procrustes superimposition the eight shape coordinates also show nonzero correlations (correlations up to about +/−0.5). These correlations necessarily occur as there are more variables than degrees of freedom for shape or form. Put more geometrically, shifting one landmark would affect more than one interlandmark distance. In fact, it is geometrically impossible to alter just one of the six distances while keeping all others constant. Likewise, the Procrustes shape coordinates are all geometrically linked. For example, moving one landmark away from the centroid necessitates moving the other landmarks closer to the centroid in order to keep the centroid size constant (and similarly for location and orientation). Therefore, it is geometrically impossible for interlandmark distances and shape coordinates to have only zero covariances. Minimizing bending energy during the sliding landmark algorithm further introduces covariances between the shape coordinates (e.g., Cardini, 2019).

The second reason is a biological one: it is not clear what a completely nonintegrated organism would look like; this would be incompatible with life in higher organisms. Genetic and epigenetic effects in early embryonic stages can affect many cells, and several circulating hormones control prenatal and postnatal growth of basically all body parts. Hence, most dimensions of an organism show some degree of integration, but certain parts might nonetheless be characterized by tighter developmental interactions and more local growth factors as compared with other parts. In other words, ontogenetic development imposes a hierarchy or “palimpsest” (Hallgrímsson et al., 2009) of variational factors with different and overlapping spatial scales, including local ones, but not a simple parcellation of the organism into distinct nonoverlapping variational units, not even a strictly modular genotype–phenotype map (Mitteroecker, 2009; Pavličev & Wagner, 2012). Uncovering these local or “modular” growth patterns first requires the modeling of more global growth factors and interactions (Mitteroecker & Bookstein, 2007). Terentjev (1931) and Wright (1932) modeled the covariance structure of selected length measurements of frogs and chicken, respectively, by first estimating large‐scale factors and then estimating local factors from the residual covariances. Mitteroecker and Bookstein (2007, 2008) expanded on these approaches and linked them to modern multivariate methods, but the current morphometric literature is still dominated by the interpretation of raw covariances or correlations in the tradition of Olson and Miller (1958), even for shape coordinates. We disagree with this practice.

These studies typically also fail to account for differences in variance between groups that inevitably affect the detected magnitudes of covariance and correlation. For instance, some studies reported higher “integration” (i.e., covariance) in upper and lower jaws in chimpanzees as compared to humans. But clearly, the developmental and mechanical mechanisms of integration are the same in both species, chimps just vary much more in their degree of prognathism, which also inflates the covariances (e.g., Mitteroecker et al., 2012).

A central problem in this context is the ignorance of spatial autocorrelation among morphometric measurements (Bookstein, 2015; Gonzalez et al., 2019; Mitteroecker & Bookstein, 2007). Adjacent parts of an organism cannot vary independently in their dimensions, just for spatial reasons. Hence, a set of adjacent measurements is expected to show higher covariances than more distant traits, even in the absence of specific modular factors. This “rule of neighborhood” (Lewenz & Whiteley, 1902) can account for most findings of “modularity” in the morphometric literature as they usually report spatially contiguous modules, typically along the most elongated axis of the studied anatomy (Mitteroecker, 2009). For our face data, for instance, the correlations between shape coordinates strongly relate to the average distance between the landmarks (*r* = −0.65, *r* = −0.66, and *r* = −0.24 for the x, y, and z coordinates, respectively). This raises the question as to what *is* a good null model in geometric morphometrics. The Mardia‐Dryden distribution of exactly uncorrelated landmark variation may sufficiently idealize independent measurement error, but not any realistic biological variation because it does not account for spatial autocorrelation. Contrary to our biological intuition, closely adjacent landmarks show the same amount of variance and covariance as distant landmarks under the Mardia‐Dryden distribution. In other words, the variation of, say, four adjacent landmarks and four distant landmarks is the same, but if we scale them all to the same centroid size, we would find that the smaller landmark configurations show more shape variance than the larger configurations^9^: shape variance actually decreases with spatial scale in the Mardia‐Dryden distribution.

**FIGURE 10:**
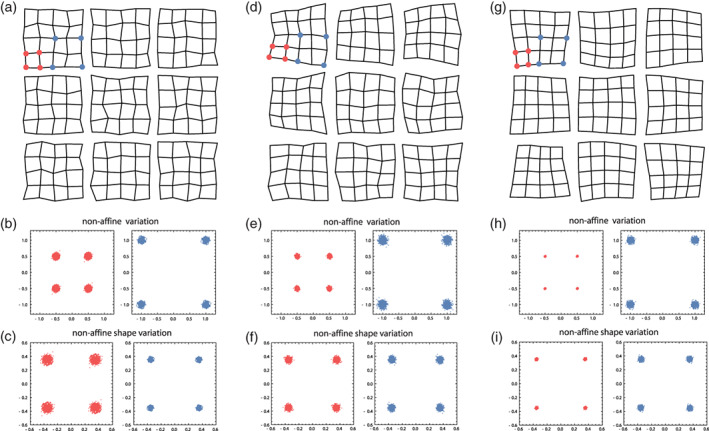
Examples of configurations of 25 landmarks each, arranged as the vertices of 5 × 5 grids. In (a) the landmarks follow a Mardia‐Dryden distribution, that is, all coordinates vary independently and with the same variance. (b) For 100 configurations drawn from this distribution, the landmark variation is shown for the vertices of a small (1 × 1) and a larger (2 × 2) grid element (red and blue points, respectively). The coordinates of both configurations show the same variance, but when scaled to the same size (c), the non‐affine shape variance is lower for the large configuration: Shape variance decreases with spatial scale. The spaces in‐between the landmarks (the 1 × 1 grid cells) thus show a tendency to “compensate” adjacent shape variation. (d) Here, the landmarks follow a self‐similar shape distribution (see main text). Every grid cell, regardless of its size, has the same non‐affine shape variation (e, f), which implies that the landmark coordinates show a pattern of spatial autocorrelation. (g) The grid cells show coordinated variation, that is, a positive correlation among adjacent cells, which is the opposite scenario as in panel a. Here, shape variance increases with spatial scale (h, i). *Source*: modified from Mitteroecker et al., 2020

This fallacy led Kanti Mardia, Fred Bookstein, and colleagues (Bookstein, 2015; Mardia et al 2006) to propose another distribution, the so‐called self‐similar shape distribution, where the non‐affine shape variance is the same at every spatial scale (hence the term “self‐similar”). This distribution does show a spatial autocorrelation: the covariance among landmarks decreases with their spatial distance. The two distributions thus differ fundamentally in their biological interpretation. Under the Mardia‐Dryden distribution, the landmark coordinates vary independently and isotropically, whereas under the self‐similar distribution the elements of the spaces *in‐between the landmarks* vary independently and isotropically (Figure 10). This can be interpreted as an idealized model of completely irregular and uncoordinated tissue growth: independent variation in the shape of constituting elements (e.g., cells or bones), as opposed to independent variation in the landmarks on the borders between elements. Clearly, this is also not how higher organisms grow, but it serves as a null‐model where no particular growth regulation occurs.

The self‐similar shape distribution is also interesting as it differentiates between two alternative scenarios. If non‐affine shape variance decreases with spatial scale, larger structures are more canalized in their shape variance than smaller substructures. This would reflect *compensatory* behavior of the underlying tissue (sensu Mitteroecker et al., 2020; Bookstein, 2015, referred to this as “disintegrated” shape variation). For instance, if one element shows a particularly strong elongation in one direction, the adjacent elements would compensate for this by reduced elongation along this direction, and vice versa for a particularly weak elongation. Despite considerable variation of every element, these elements *together* (i.e., at a larger scale) would then show only little variance. In the opposite scenario, the elements show a similar behavior, for example, if one element shows an elongation along one direction, the adjacent elements tend to have a similar elongation. Mitteroecker et al. (2020) referred to this as *coordinated variation*, which leads to an increase of non‐affine shape variance with spatial scale, that is, larger structures tend to be more variable in shape than smaller structures.

In practice, these behaviors can be assessed by plotting the variance of partial warp scores against the inverse of bending energy as a measure of spatial scale (Bookstein, 2015, 2018; Mitteroecker et al., 2020). Under a self‐similar shape distribution, non‐affine shape variance increases linearly with spatial scale. Regression of partial warp variance on the inverse of bending energy at a log–log scale thus yields a slope of 1 for 2D landmarks and of 2 for 3D landmarks, respectively. Coordinated variation, by contrast, leads to a greater regression slope and compensatory variation to a smaller slope. As a rough surrogate, one can also plot the non‐affine shape variance of different landmark configurations (different substructures, e.g., the different bones of the cranium) against their squared average centroid size. An increase of non‐affine shape variance with squared centroid size would indicate coordinated variation and a decrease compensatory variation. Exceptions of some structure or partial warps from such a general scaling trend may indicate some deviant local processes. For instance, Mitteroecker et al. (2020) found that in the human cranium the shape variance clearly decreases with spatial scale, that is, the shape of the separate cranial bones varied much more than overall cranial shape. This indicates that the cranial bones tend to compensate for deviant shapes of the adjacent bones in order to guarantee a “normal” shape of larger, functional cranial units. Only the nasal bones clearly deviated from this pattern.

The separation into small‐scale and large‐scale shape variance can also be useful for evolutionary and phylogenetic studies. Small‐scale features, such as the shape of separate bones, often are of less functional importance than large‐scale features. For instance, the overall dimensions of the jaws are clearly functionally relevant and thus under natural selection, but the relative size and shape of the premaxilla, maxilla, and palatine are of little functional importance as long as the overall jaw dimensions are functional. These small‐scale features may thus be more strongly subject to evolutionary drift across species and reduced stabilizing selection within species as compared with large‐scale features. Accordingly, Grunstra et al. (2021) found that small‐scale cranial shape features better reflect papionin phylogenetic history than large‐scale features, whereas individuals better clustered into species for the large‐scale shape features.

In another application, Windhager et al. (2017) studied the spatial scale of human facial shape features associated with specific body characteristics and trait attributions during social perception. They found that physiological characteristics were represented at larger scale, whereas cues in perception were mainly driven by small‐scale shape features: raters relied on small‐scale features in the face when judging health status, as opposed to the large‐scale shape patterns that relate to hormone levels and body mass index. These insights facilitate studies on an additional range of traits, such as facial ratings of emotions, which may correspond to facial features at even smaller scales.

In contrast to studies that aim to identify modules, purely exploratory studies of morphological integration, typically performed by partial least squares analysis (Adams & Felice, 2014; Baab, 2013; Bookstein et al., 2003; Klingenberg & Zaklan, 2000; Mitteroecker & Bookstein, 2007; Rohlf & Corti, 2000), are unaffected by the geometric and biological caveats mentioned above; geometric dependences and spatial autocorrelations are simply part of the assessed associations. This way, numerous studies have reported patterns of integration in human and primate anatomy and inferred evolutionary constraints or drives from these patterns (e.g., Bastir et al., 2006, 2010; Mitteroecker & Bookstein, 2008; Neaux et al., 2019; Singh et al., 2012).

## 10 STUDYING ASSOCIATIONS BETWEEN ORGANISMAL FORM AND OTHER VARIABLES

Many morphometric studies are concerned with relating organismal form to other variables, such as environmental, functional, behavioral, or genetic data. Because of the inherently multivariate nature of geometric morphometrics, such associations cannot be studied variable by variable. One standard approach is to regress *all* shape or form coordinates onto one external variable and to visualize the resulting vector of regression coefficients as a shape or form deformation (“shape or form regression”). For example, when regressing the shape coordinates on body mass index as in Figure 7, the regression slopes represent the average shape change that is linearly associated with one unit change in BMI. For the sake of effective visualization, a multiple of these coefficients are added to the mean shape to achieve an interpretable deformation. When regressing shape on the genotype score of an allele at one genetic locus (i.e., the count of this allele at this locus: 0, 1, or 2), the slopes represent the average genetic effect resulting from one allele substitution (Mitteroecker et al., 2016).

When the external variables are also numerous and may better be interpreted in terms of linear combinations of the original measurements, we need methods that treat both blocks of variables in a multivariate way. Partial least squares analysis has mostly been used for this purpose in geometric morphometrics (e.g., Baab, 2013; Bookstein, 1991; Bookstein et al., 2003; Mitteroecker & Bookstein, 2007; Rohlf & Corti, 2000; Windhager et al., 2011). However, at least three different principal approaches are possible within the classic multivariate linear framework. These methods generalize the three bivariate statistics, covariance, regression, and correlation, to “latent variables” that are estimated from the multivariate measurements. The underlying rationale is that we have measured many variables but assume that variation in these many variables is driven by only a few unmeasured factors, the latent variables. For instance, we have measured many climate variables, but we assume that only one or a few climate properties affect organismal form across populations. As we do not know these properties, we do not know which of our measures, or combination of measures, best describe these properties. Thus, we try to estimate these properties—the latent variables—as linear combinations of the measured variables. The same holds for the morphometric side. We do not assume that climate affects all aspects of organismal form; only a few specific features (the latent form variables) are responsive to these environmental factors. As we aim for a comprehensive representation of the association between form and climate, we estimate the latent variables as linear combinations that *maximize* the association between the latent variables of form and climate. Here one can choose among the three standard measures of linear association to be maximized.

The covariance measures the joint variance of two variables, whereas the squared correlation is the fraction of variance in one variable that is explained by the linear association with the other variable. The correlation is equivalent to the covariance of the two *z*‐transformed variables (i.e., scaled to unit variance); the correlation coefficient thus is scale invariant. Both statistics are symmetric. The regression slope, by contrast, measures the average change of the dependent variable associated with one unit change of the independent variable. Seeking a pair of latent variables, one for each block of variables, that maximizes the *covariance* between these latent variables is equivalent to two‐block partial least squares analysis (PLS). Maximizing the *regression slope* of the dependent latent variable on the independent latent variable is equivalent to reduced rank regression (RRR; Aldrin 2000; Izenman 1975), whereas maximizing the *correlation* between the latent variables is achieved by canonical correlation analysis (CCA). Computationally, all three methods are based on a singular value decomposition, but of different matrices (see, e.g., Mitteroecker et al., 2016, and Stansfield et al., 2021, for computational details). Therefore, all three methods yield multiple pairs of latent variables with successively lower covariance, regression slope, or correlation, respectively.

When should we choose which method? PLS is computationally most stable as it does not require an inversion of a covariance matrix. RRR involves an inversion of the covariance matrix of the independent variables and thus requires manifold more cases than independent variables (Section 5). CCA has the same requirement even for both blocks of variables. In practice, CCA thus requires dimension reduction or a regularization of the covariance matrices prior to the actual analysis. In contrast to RRR, the results of PLS are strongly influenced by the variance of the variables. Consider, for instance, measures of minimum and maximum temperature as well as some shape variables. Let us assume that both temperature variables are highly variable and correlated; the *difference* between maximum and minimum temperature thus varies little. Assume further that some aspects of shape are weakly affected by maximum and minimum temperature, whereas other shape features are highly responsive to the difference between maximum and minimum temperature. In other words, the average temperature would have a high covariance with shape, but the regression slope would be small (one unit temp. has a small average effect on shape). By contrast, the temperature difference would show less covariance with shape because it varies just little in the sample, but the regression slope would be high, reflecting the strong average impact of one unit temperature difference on shape. Accordingly, the first dimension of PLS, which maximizes the covariance, would represent the average temperature (similar loadings for both measures), but RRR would find the temperature difference (positive loading for max. temp. and negative loading for min. temp., or vice versa) as first latent variable because it maximizes the regression slope. For CCA another criterion matters, namely additional factors of variation that are not accounted for by the measured variables (the unexplained variance). As CCA maximizes the explained variance, it will find latent variables for which this unexplained variance is a minimum, for example, shape features that are mostly determined by temperature, even if weakly, but not by any other environmental or genetic factors. Clearly, it depends on the research question if one wants to identify shape features that covary most with temperature in the sample, that are most responsive to temperature regardless of the actual (co)variance, or that are most predictable by temperature.

Published applications of PLS and RRR include the search for geographic clines in shape. For example, Frost et al. (2003) used a PLS between cranial shape variables and geographic coordinates (latitude and longitude) for different papionins, while Grunstra et al. (2018) used a RRR for similar data. The loadings for the geographic coordinates could directly be interpreted as a geographic direction along which the shape features depicted by the corresponding loadings are changing in the sample. Here, PLS yields the geographic direction with the strongest covariance with shape, but RRR gives a direction along which one unit change (e.g., 1 km) has the strongest average impact on shape. The results would differ among the two methods if along one geographic direction the sample has its largest geographic extension and thus most variance in the coordinates, but another direction shows the strongest cline, that is, the strongest effect on shape per km. CCA, by contrast, yields the direction for which some shape features are most predictable by geographic location (least unexplained variance).

If one block of variables consists of genotype scores for different loci, as in genetic association studies, RRR yields genetic latent variables with the strongest average genetic effect on the phenotypic latent variable, whereas CCA leads to latent variables with the highest heritability (Mitteroecker et al., 2016). PLS leads to latent variables that covary most in the sample, regardless of the average effect and heritability. For instance, an allelic pattern that varies strongly in the sample might be revealed by PLS even if the average genetic effect on the phenotype is limited. RRR, by contrast, yields an allelic pattern with strong effects on the phenotypic latent variable per allele substitution, even if they vary little in the sample.

**FIGURE 11:**
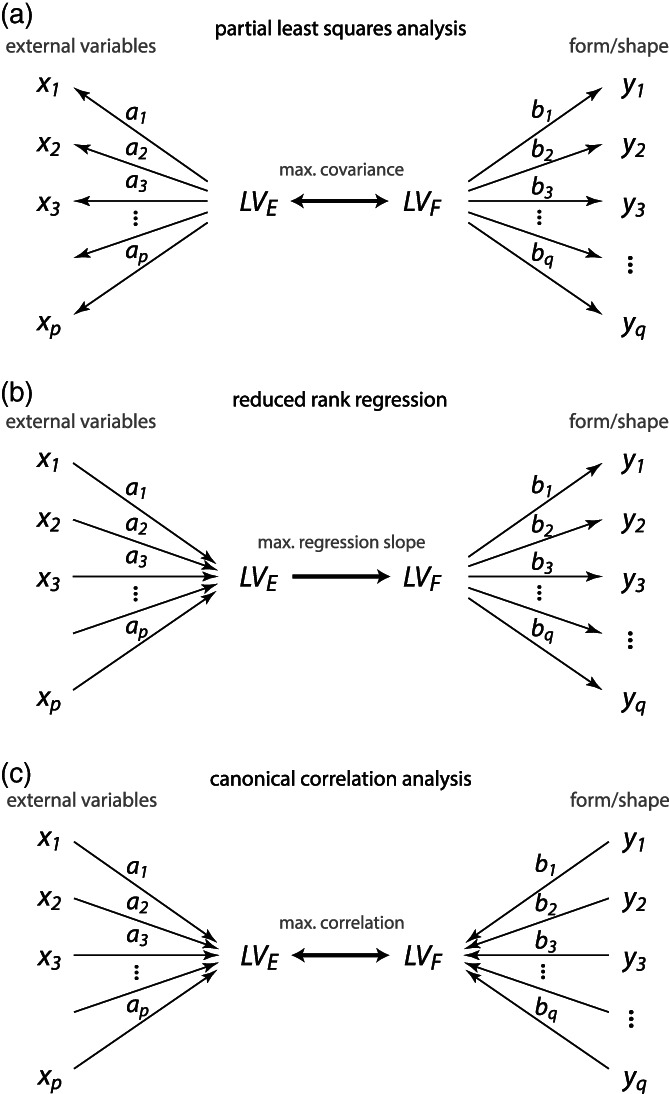
Path models illustrating two‐block partial least squares analysis (PLS), reduced rank regression (RRR), and canonical correlation analysis (CCA). In all three methods, the multivariate association between two blocks of measured variables (e.g., one block of shape variables and one block of environmental variables) is decomposed into multiple pairs of latent variables. (a) In PLS, both blocks of variables are treated symmetrically, and the latent variables are estimated as linear combinations of the variables with maximal covariance. The loadings or coefficients are proportional to covariances or multivariate regression coefficients between the measured variables and the latent variables. (b) RRR maximizes the regression slope of the dependent latent variable on the independent latent variable. The coefficients of the independent variables are proportional to multiple regression coefficients on the corresponding latent variables, whereas the coefficients of the dependent variables are proportional to covariances. (c) CCA maximizes the correlation between the latent variables, and the coefficients are proportional to multiple regression coefficients for both blocks

Moreover, the interpretation of the loadings for the two blocks of variables differs somewhat among the three methods. In PLS, the coefficients for the measured variables are proportional to the regression coefficients of the variables on the corresponding latent variable (Figure 11a). In RRR, by contrast, the coefficients for the independent variables are proportional to the *partial* regression coefficients of the latent variable on the measurements (Figure 11b). The coefficients of the dependent variables are multivariate regression coefficients, just as in PLS. CCA yields partial regression coefficients for both blocks of variables (Figure 11c). These differences can be relevant if the variables are highly correlated. For instance, let us again assume we measured minimum and maximum temperature along with some shape variables. But now assume that minimum temperature alone is driving the association with shape, while maximum temperature has no effect. *In our sample*, however, the two temperature measures are highly correlated, that is, individuals experiencing high minimum temperature also tend to experience high maximum temperature, and vice versa. As a result, both variables show some covariance with shape, even though maximum temperature has no causal effect on shape. However, in a multiple regression of the shape feature (latent variable) on both temperature measures, only the partial regression coefficient for minimum temperature would reflect an effect while that for maximum temperature would be close to zero (conditioned on min. temperature, max. temperature has no association with shape any more). In PLS, therefore, both temperature variables would have high coefficients, whereas in RRR and CCA maximum temperature would have a coefficient close to zero.

In summary, the properties and interpretations of RRR most closely resemble the typical scientific questions in anthropology and biology, but PLS is computationally more convenient as it does not necessarily require prior variable reduction. PLS is also computationally less demanding than the other methods, which can matter in genetics and other fields, where the number of independent variables is often huge. CCA may find applications in forensics and image recognition, where prediction and classification are the main tasks, but computationally it is the least stable approach.

## 11 OUTLOOK: AUTOMATED LANDMARKING AND LANDMARK‐FREE APPROACHES

The manual setting of landmarks is a time‐consuming process that requires anatomical background knowledge. It is also prone to error (e.g., Fruciano, 2016; Menéndez, 2017; Waltenberger et al., 2021). In the last few years, numerous algorithms and software implementations for automated landmarking have been published (Aneja et al., 2015; Bannister et al., 2020; Bromiley et al., 2014; Devine et al., 2020; Galvánek et al., 2015; Le et al., 2020; Li et al., 2017; Percival et al., 2019; Porto et al., 2021; Porto & Voje, 2020; Vandaele et al., 2018). Personally, we have no experience with these approaches, but the publications and what we have heard from colleagues appear promising. However, these methods do not seem to work for all anatomical structures equally well, and occasional misplacements of landmarks do occur. Testing these algorithms on small samples prior to actual data collection as well as careful control of automatically placed landmarks seems advisable.

Also, a large number of different “landmark‐free” or “homology‐free” morphometric approaches have been suggested, all of which have in common the notion that no identification of homologous landmarks is required. The simplest class of these methods is based on some automated placement of points on curves or surfaces, such as the iterative closest point algorithm, followed by multivariate analysis of the registered point coordinates as in geometric morphometrics (e.g., Gonzalez et al., 2016; Polly, 2008; Pomidor et al., 2016) or in terms of radii or angular changes along outlines or surfaces (e.g., eigenshape analysis; MacLeod, 1999; Polly & McLeod, 2008). In another class of methods, curves or surfaces are represented by some continuous functions, such as sine and cosine functions (elliptical Fourier analysis and spherical harmonics; e.g., Shen et al., 2009; Caple et al., 2017), but also other approaches have been suggested (e.g., Joshi et al., 2010). In recent years, deformation‐based methods and diffeomorphometry have become more common in anthropology and evolutionary biology (e.g., Bône et al., 2018; Boyer et al., 2011; Durrleman et al., 2012, 2014; Koehl & Hass, 2015; Rolfe et al., 2011; Specht et al., 2007; Toussaint et al., 2021; Urciuoli et al., 2020). These methods originate in medical image analysis, especially brain imaging (Ashburner et al., 1998). Most of these methods make use of registered surface points (“control points”) and an estimate of the sample mean shape or some other reference shape (“atlas”). The core idea is that the shape differences between each object and the reference shape are represented as deformations (diffeomorphic or conformal maps, i.e., invertible “smooth” maps from one smooth surface to another). Via a kernel function, one can determine the spatial resolution of these deformations. The parameters of the deformations are then used for multivariate statistical analysis. Unlike in geometric morphometrics, however, the ensuing shape space is a highly nonlinear manifold that requires nonlinear statistics, such as geodesic distances, Fréchet means, geodesic regressions, and nonlinear dimension reduction.

The proponents of these methods are (naturally) optimistic about their performance, and the published studies indeed appear promising. However, the complete loss of point homology (e.g., landmarks on sutures that represent the contribution of different bones) and the non‐Euclidean nature of shape space can challenge some of the typical inferences in morphometrics way beyond the extent described in the previous sections. Consider, for instance, two faces that differ in the position of the nose. If the tip of the nose is represented by the *same* homologous landmark in both faces, the average face has a nose with an average position. But if the tip of the nose is captured by different points in the two faces (as in landmark‐free morphometrics), the analysis will correctly identify differences in face shape, but the mean of those shapes is not a proper face anymore because the computed average nose is a composite of the two noses in different positions (see also Klingenberg, 2008). Likewise, if we study the variance in face shape without point homology, we would not find the maximum variance to be located in the position of the nose but in the areas at the edge of the average nose, which in some individuals belongs to the nose and in some individuals not. Despite this problem, cluster analysis and statistical classification (the typical tasks in medical imaging) can still be successful, but other biometric analyses are difficult. This situation can also be considered as a non‐Euclidean shape space: not all possible point configurations or deformations yield realistic face shapes. The space of actual face shapes is a non‐Euclidean subspace (a manifold) embedded within the space of all shapes. In order to transform a face into another, one needs to find the shortest path along this curved manifold of face shapes (the geodesic or Riemannian distance), but depending on the actual parametrization of face shape and facial deformations, there can be many such possible paths and many possible intermediate shapes. Similarly, applying a shape deformation (e.g., a smile) from one face to another face requires one of multiple possible “transport” operations (e.g., Piras et al., 2021), whereas in geometric morphometrics a vector of shape change can simply be applied to another shape (e.g., in “developmental simulations”; McNulty et al., 2006; Neubauer & Gunz, 2018). This challenges the interpretation of linear shape trajectories and the notion of “morphological intermediacy,” that is, all the affine invariant geometries described in Section 5. For instance, if phenotypes that are heterozygous for a given genotype are located in shape space at the midpoint of the homozygous phenotypes, this is interpreted as the result of purely additive allele effects (codominance), whereas deviations from the midpoint indicate non‐additive allele effects (Klingenberg et al., 2001; Pavličev et al., 2016). Similarly, two parallel linear trajectories in shape space reflect two identical and continual shape transformations applied to different starting shapes (e.g., Mitteroecker et al., 2005). It still remains to be shown if similar interpretations are warranted for landmark‐free morphometric approaches.

In summary, the utility of landmark‐free morphometric methods for biometric analyses beyond mere discrimination and classification has still to be explored. The combination of homologous point locations with semilandmarks on curves and surfaces in geometric morphometrics enables a wide range of analyses and biological interpretations. Perhaps, the costs (in terms of biometric interpretability) of discarding point homology are too high, and, thus, the geometric morphometric framework combined with automated landmarking is a safer way into the next decades of morphometrics.

## 12 CONCLUSIONS

Much has been achieved since the “revolution” in morphometrics. An ever‐growing community has been advancing and applying geometric morphometrics within biology, anthropology, archaeology, and numerous related fields. Improved imaging technologies have extended geometric morphometric analyses to complex 3D structures, to microscopic and embryonic scales, to precious fossil and archeological specimens, and also to 3D surface scans and medical images of living people. Yet, the large number of landmarks and semilandmarks that came along with these developments can challenge some statistical analyses and—much more so—some of the biological interpretations. Classic biometrics and quantitative genetics are based on measured “traits,” that is, discernable and homologous biological characteristics that can be separately represented by a single variable each. All the classic concepts of morphological integration rest on such measured traits. But the data generated in geometric morphometrics are fundamentally different. None of the landmark coordinates can be interpreted separately; all analyses are multivariate and generate shape or form features that combine many, if not all of the measured landmark coordinates. Insights on “biological homology” can emerge in the course of the analysis and are not necessarily a simple a priori of the measured landmarks. Single scalar summary statistics and *p*‐values thus are generally less insightful than the exploration or “calibration” of shape features that align with hypothesized or measured biological factors. Modern multivariate statistics and machine learning will certainly resolve most of the technical difficulties currently encountered in geometric morphometrics, but the conceptual and biometric challenge to profoundly connect geometric morphometrics to developmental, evolutionary, and behavioral biology cannot be solved by computational scientists. It will require the continuing efforts of the morphometricians amidst all these disciplines, as well as another generation of well‐prepared students, to tackle these fascinating problems.

## CONFLICT OF INTEREST

The authors have no conflicts of interest to report.

## Supporting information


**Supplementary Figure S1** Two different principal component analyses of the midsagittal cranial shape of a modern chimpanzee, an Australopithecus (STS 5), and a Middle Paleolithic human fossil (Kabwe 1). The landmarks are taken from Bookstein et al. (2003). In (a) the neurocranium is covered by a much denser set of landmarks and semilandmarks as compared with (b), but the first principal component (PC 1) corresponds to a very similar shape deformation in both analyses: facial projection, basicranial flexion, and neurocranial globularity. However, the position of STS 5 along PC1 differs among the two landmark schemes. In (a) it is closer to Kabwe whereas it is closer to the chimp in (b). This discrepancy results from the different relative weighting of neurocranial shape through the different number of neurocranial landmarks. With fewer neurocranial landmarks, the facial similarity among STS 5 and the chimp dominates the overall shape ordination, whereas the larger neurocranial landmark set brings STS 5 closer to Kabwe because of neurocranial similarities (especially in the occipital).

## Data Availability

The ALSPAC study website contains details of all the data that are available through a fully searchable data dictionary (www.bris.ac.uk/alspac/researchers/data-access/data-dictionary/) and will be made available on request to the Executive (alspac‐exec@bristol.ac.uk).

## Endnotes

^1^
An affine transformation is a geometric transformation that preserves lines and parallelism. In other words, affine shape transformations consist only of translation (moving the object) and linear scaling along one or more axes (stretching, shearing). A deformation grid that captures such an affine shape difference would only consist of straight and parallel lines (hence zero *bending* energy), and the grid cells would all have the same form. The shape difference thus is not localized; it is the same everywhere. For non‐affine shape differences, by contrast, the grid cells differ in form; the shape difference has a location and a certain spatial scale, which is inversely related to bending energy (the smaller the deformation, the more are the grid lines bent).

^2^
More specifically, Procrustes or Kendall shape space is not a Euclidean (i.e., “linear”) space but a Riemannian manifold (a “smoothly curved” space) of dimension 2*k*‐4 or 3*k*‐7 for two‐ or three‐dimensional landmarks, respectively (Bookstein, 1996; Dryden & Mardia, 1998; Kendall, 1984). For instance, *k* 2D landmarks have 2*k* coordinates with only 2*k*‐4 degrees of freedom due to the standardization of variance in overall location, scale, and orientation of the configurations. In other words, within the 2*k*‐dimensional Euclidean space of the variables, the shapes are points located within a curved subspace of dimension 2*k*‐4. But in almost all geometric morphometric applications, the shape variance is small enough to ignore or remove (through tangent space project) this curvature and to treat it as linear (Marcus et al., 2000; Rohlf, 1999). For our face data, the curvature of shape space accounted for only 0.02% of total variance, which was in the order of the variance of PC 75. In most geometric morphometric applications, the curvature of shape space thus is irrelevant, and we will not treat it further here. However, in other branches of shape analysis, for example, deformation‐based morphometry, the curvature of shape space can be massive and linear statistics cannot by applied (see Section 11).

^3^
If, in the simplest case, a 2D data space had two non‐orthogonal axes, moving a data point along one axis automatically alters the other coordinate, too. Only for orthogonal axes, one coordinate can be changed without changing the other coordinate: This implies that the two variables are geometrically independent, that is, they *can* vary independently. Note that geometric independence does not imply statistical independence. But only if the variables are geometrically independent, can a correlation of zero be interpreted as statistical independence.

^4^
Note that here the term “affine transformation” (i.e., linear scaling and translation) refers to a transformation of the 2*k* or 3*k*‐dimensional data space, or a low‐dimensional ordination of it, not the 2D or 3D ambient space (as in note 1).

^5^
This is reflected, for instance, by the well‐known chi‐squared distribution, the distribution of the squared distance from the mean in a one‐ or higher‐dimensional normal distribution. For a single normal variable, most of the cases are close to the mean, and the average squared distance from the mean is small. For two variables, the average multivariate distance from the mean is larger because we need to add the squared distance from the mean along both variables, and so on. In fact, the expected value of a chi‐squared distribution, that is, the average squared distance from the mean, is equal to the number of variables. In other words, in high‐dimensional space, all points are far apart from the mean.

^6^
These are well‐known problems in statistics and machine learning, for which multiple methods have been suggested, such as ridge regression, LASSO, or latent‐variable models (see, e.g., Johnstone & Titterington, 2009, for an overview). These methods, however, do not overcome the problems of interpreting distances and angles in high‐dimensional spaces.

^7^
A data matrix has full rank if all its columns are linearly independent, that is, if none of the variables can be exactly predicted by the other variables. This implies that all the principal components have nonzero variance; only then can a covariance matrix be inverted. In practice, rank deficiency arises if *p*≥*n* and from variable standardization. Due to the standardization for position, size, and orientation, Procrustes shape coordinates are not of full rank.

^8^
To wrap up the somewhat challenging terminology: “Relative warps” are principal components of shape or form coordinates, that is, shape features of successively smaller variance (eigenvectors of the corresponding covariance matrix). Relative warps can be weighted by bending energy to up‐ or down‐weight components of large or small scale (the parameter α in Bookstein, 1991 and Rohlf, 1993). “Principal warps” are non‐affine shape features of successively larger spatial scale (the eigenvectors of the bending energy matrix; Bookstein, 1991, 2018; Rohlf et al., 1996). “Partial warps” are the individual scores of the shapes along the principal warps. Relative intrinsic warps, a newer concept, are shape features with successively smaller variance *relative* to the corresponding bending energy as a measure of spatial scale (the eigenvectors of the covariance matrix relative to the bending energy matrix; Bookstein, 2018).

^9^
This is because we increase the scale of the small configurations and/or decrease the scale of the large configurations to bring them all to the same size. Hence, if they had the same variance before scaling, the small configurations have increased variance and/or the large configurations decreased variance after scaling (see Figure 10).
