# Supplementary material for: Thirty years of geometric morphometrics: Achievements, challenges, and the ongoing quest for biological meaningfulness
Source: Am J Biol Anthropol. 2022 May 29;178(Suppl 74):181–210. doi: 10.1002/ajpa.24531 (PMC9545184; doi:10.1002/ajpa.24531)
Supplement: Supplementary file 1 — Supplementary Figure S1 Two different principal component analyses of the midsagittal cranial shape of a modern chimpanzee, an Australopithecus (STS 5), and a Middle Paleolithic human fossil (Kabwe 1). The landmarks are taken from Bookstein et al. (2003). In (a) the neurocranium is covered by a much denser set of landmarks and semilandmarks as compared with (b), but the first principal component (PC 1) corresponds to a very similar shape deformation in both analyses: facial projection, basicranial flexion, and neurocranial globularity. However, the position of STS 5 along PC1 differs among the two landmark schemes. In (a) it is closer to Kabwe whereas it is closer to the chimp in (b). This discrepancy results from the different relative weighting of neurocranial shape through the different number of neurocranial landmarks. With fewer neurocranial landmarks, the facial similarity among STS 5 and the chimp dominates the overall shape ordination, whereas the larger neurocranial landmark set brings STS 5 closer to Kabwe because of neurocranial similarities (especially in the occipital). [file AJPA-178-181-s001.docx]

**Supplementary Figure 1.** Two different principal component analyses of the midsagittal cranial shape of a modern chimpanzee, an Australopithecus (STS 5), and a Middle Paleolithic human fossil (Kabwe 1). The landmarks are taken from Bookstein et al. (2003). In (a) the neurocranium is covered by a much denser set of landmarks and semilandmarks as compared with (b), but the first principal component (PC 1) corresponds to a very similar shape deformation in both analyses: facial projection, basicranial flexion, and neurocranial globularity. However, the position of STS 5 along PC1 differs among the two landmark schemes. In (a) it is closer to Kabwe whereas it is closer to the chimp in (b). This discrepancy results from the different relative weighting of neurocranial shape through the different number of neurocranial landmarks. With fewer neurocranial landmarks, the facial similarity among STS 5 and the chimp dominates the overall shape ordination, whereas the larger neurocranial landmark set brings STS 5 closer to Kabwe because of neurocranial similarities (especially in the occipital).
